# Navigating strategies for intercultural maternal and newborn care in Latin America and the Caribbean: a scoping review

**DOI:** 10.1093/heapro/daag082

**Published:** 2026-06-15

**Authors:** Mylena Maria Guedes de Almeida, Thiago Barreto do Nascimento Filho, Renata Isadora Ruiz Cortés, Fiorella Iturrino Vilchez

**Affiliations:** Escola Paulista de Medicina, Universidade Federal de São Paulo, Rua Botucatu, 740, Vila Clementino, São Paulo 04023-062, Brazil; Faculdade de Medicina, Universidade Tiradentes, Av. Murilo Dantas, 300, Farolândia, Aracaju 49032-490, Brazil; Faculty of Social Sciences, Charles University, Smetanovo nábř. 6, Staré Město, Prague 100 00, Czech Republic; Faculty of Social Sciences, Charles University, Smetanovo nábř. 6, Staré Město, Prague 100 00, Czech Republic; Escuela de Gobierno y Políticas Públicas PUCP, Pontifical Catholic University of Peru, Complejo Mac Gregor, Av. Universitaria 1801, San Miguel 15088, Lima, Peru

**Keywords:** maternal health, intercultural care, cultural competence, global health, health policy

## Abstract

In Latin America and the Caribbean (LAC), women from racial and ethnic minority groups experience disproportionately worse maternal health outcomes. In addition to facing barriers to accessing maternity services, they often receive culturally inappropriate care. To address these inequities, a range of intercultural approaches to maternal and newborn care has been implemented across the region. However, the extent and nature of these initiatives over the past decade remain underexplored. We conducted a scoping review to identify, categorize, and describe strategies for promoting intercultural maternal and newborn care in LAC. We searched nine databases and grey literature for studies published in English, Spanish, or Portuguese between 2013 and 2024. This review identified 75 studies and 16 strategy categories across 13 countries. Most studies addressed Indigenous populations and employed qualitative methods. Strategies were grouped into four cross-cutting domains: (i) healthcare adaptations and workforce capacity building, (ii) intercultural communication and knowledge exchange, (iii) community engagement, and (iv) institutional support and governance. The identified strategies engaged stakeholders at the community, facility, and system levels. Based on these findings, we developed a framework for interculturality in maternal and newborn care. Our analysis indicates that sustainable implementation depends on addressing structural barriers and integrating intercultural principles into health system planning, service delivery, policy, and governance. The proposed framework can inform research, policymaking, and development of intercultural maternal and newborn care initiatives in LAC and other culturally diverse settings.

## Graphical Abstract

**Graphical Abstract:**
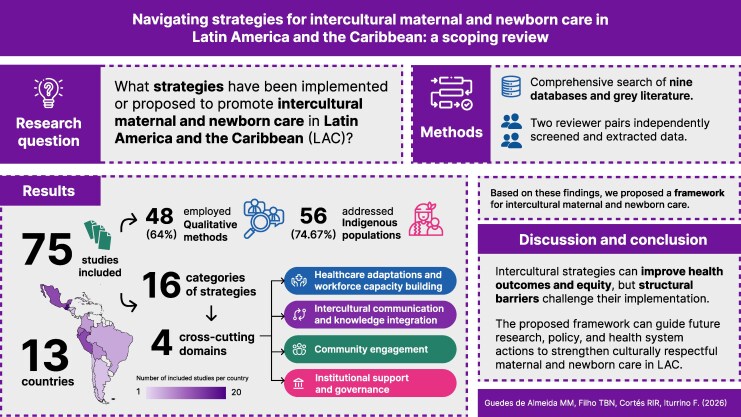

Contribution to Health Promotion

- Intercultural maternal and newborn care strategies are increasingly recognized as essential for improving health outcomes in Latin America and the Caribbean, although the range and scope of strategies across the region remain largely unmapped.
- By synthesizing evidence from 13 countries, this review identified 16 categories of intercultural maternal and newborn care strategies and proposed a framework to guide policy and practice.
- The identified strategies connect interculturality in maternal and newborn care with core health promotion action areas, including community empowerment, workforce capacity building, and the reorientation of health services towards equity, dialogue, and supportive environments for diverse cultural groups.

## Introduction

Sustainable Development Goal (SDG) 3.1 aims to reduce the global maternal mortality ratio (MMR) to less than 70 deaths per 100 000 live births by 2030. However, current trends suggest the world is not on track to meet this target (World Health Organization 2023). In 2020, the global MMR was estimated at 223, with over 90% of maternal deaths occurring in low- and middle-income countries (LMICs), many of which have experienced long-standing stagnation in reducing the MMR (World Health Organization 2023, Souza *et al*. 2024). Efforts focused exclusively on biomedical causes are insufficient and may help explain this stagnation (World Health Organization 2023, Souza *et al*. 2024). Addressing this challenge also depends on social determinants of maternal health, which are the underlying economic, political, and cultural forces that broadly influence the health and well-being of women before, during, and after pregnancy (Souza *et al*. 2024).

Among these determinants, culture is critical but often neglected in global health policy and research (Aubel and Chibanda 2022). Leininger’s (1996) theory of culture care, widely applied in midwifery and adopted in this study, defines culture as the learned, shared, and transmitted values, beliefs, norms, and lifeways that guide thinking, decisions, and actions in patterned ways (Leininger 1996). Empirical studies consistently show that ethnic minority patients in both LMICs and high-income countries (HICs) (Degrie *et al*. 2017, Watson *et al*. 2019, Al-Mubarak *et al*. (2025) receive less and culturally inappropriate care during pregnancy, childbirth, and the postpartum period, while facing persistently high MMRs.

In Latin America and the Caribbean (LAC), these disparities are also evident (Pan American Health Organization 2017), especially for Indigenous women (Paulino *et al*. 2019, Bautista-Valarezo *et al*. 2022). Compared to the general population, they face substantial barriers to accessing adequate healthcare and experience disproportionately poorer maternal health outcomes (Paulino *et al*. 2019, Bautista-Valarezo *et al*. 2022, Sáenz *et al*. 2024). These inequities are rooted in structural violence, including racism, gender inequality, and poverty (Gamlin and Holmes 2018). In response, the concept of *interculturalidad* (interculturality) gained prominence in the 1990s, driven by Indigenous movements advocating for health rights across the region (Nureña 2009). Within this context, the Pan American Health Organization (PAHO) defines interculturality as an interactive social process of recognizing and respecting cultural differences, an essential foundation for building a fair society (Organización Panamericana de la Salud 2008). Although often used interchangeably with ‘cultural competence’, interculturality emphasizes interaction and dialogue, whereas cultural competence refers to awareness and respect (World Health Organization 2020). At the health system level, interculturality can address power asymmetries and promote co-construction of care with diverse communities (Anand and Lahiri 2009). In maternal and perinatal health, intercultural strategies have demonstrated potential to address historical discrimination by fostering trust, dialogue, and preserving cultural identities (Vivar 2007, Tubino 2016).

Strategies involving the training and integration of traditional birth attendants (TBAs) into formal health systems represent a crucial dimension of interculturality in maternity care and have been examined in several reviews across different global regions (Byrne and Morgan 2011, Wilson *et al*. 2011, Sibley *et al*. 2012, Rutledge *et al*. 2024). Additionally, Coast *et al*. (2014) conducted a systematic global mapping of interventions implemented in 35 countries up to 2013, which addressed cultural factors influencing the use of services provided by skilled birth attendants (SBAs), such as doctors and nurses. In LAC, their review highlighted adaptations in service delivery models, including permitting family presence during childbirth and establishing maternity waiting houses.

While these previous reviews have explored relevant strategies related to intercultural maternal health, neither interculturality nor LAC were their central focus. As a result, there is limited understanding of the range of strategies implemented across the region. To our knowledge, no scoping or systematic review has comprehensively examined the literature from the past decade to map and categorize strategies aimed at promoting intercultural maternal and newborn care in LAC. Moreover, existing theories on intercultural maternity care are often based on the experiences of immigrant populations in HICs and may not reflect Latin-American sociocultural contexts (Wikberg 2021). There is also a lack of integrative frameworks that bring together the diverse strategies, actors, and implementation levels involved in intercultural maternal and newborn care in the region. Therefore, a regionally focused synthesis is needed to inform policies and practices, influence advocacy efforts, workforce training, health system improvements, and community empowerment in LAC.

To address this gap, we conducted a scoping review to identify, categorize, and describe potential and existing strategies for promoting intercultural maternal and newborn care in the region and to propose a framework to support their translation into policy and practice.

## Methods

### Study design

We performed a scoping review in accordance with the recommendations of the Joanna Briggs Institute (JBI) (Peters *et al*. 2020a, 2020b). The review adhered to the PRISMA-ScR checklist (Tricco *et al*. 2018), which is provided in Supplementary File 1. The protocol was prospectively registered at https://osf.io/qsxk7 and is also included in Supplementary File 2. Ethical approval was not required because this was a secondary analysis of published data. Patients and the public were not involved in any aspect of this review.

We selected a scoping review methodology given our aim to map, identify, and report strategies within a complex and heterogeneous topic (Arksey and O’Malley 2005, Levac *et al*. 2010, Peters *et al*. 2020b). Unlike systematic reviews, which are designed to address narrowly defined questions, scoping reviews are particularly suited to broader research questions and valuable when the evidence is emerging, diverse, or fragmented (Armstrong *et al*. 2011, Peters *et al*. 2020b).

Our main research question was: what strategies have been implemented or proposed to promote intercultural maternal and newborn care in Latin America and the Caribbean? We also addressed a subquestion: what are the key characteristics of these strategies and of the studies describing or evaluating them? These questions align with core indications for conducting a scoping review: to map the available evidence and to identify key attributes associated with the concept under investigation (Munn *et al*. 2018, Peters *et al*. 2020b).

### Eligibility criteria

As recommended by JBI (Peters *et al*. 2020b), we used the PCC framework (Population, Concept, Context) to guide the development of the research question, inclusion/exclusion criteria, and search strategy.

- **P (population, condition):** Women during pregnancy, childbirth, and postpartum or neonates. Studies were excluded if they did not focus on maternal or neonatal health, for example, those addressing general adult health or child health beyond the neonatal period.
- **C (concept):** Strategies for promoting intercultural maternal and newborn care or culturally appropriate maternity care. For the purposes of this review, the term ‘strategy’ was used broadly to encompass any initiative, programme, intervention, policy action, or structured approach aimed at promoting intercultural maternal and newborn care, considering the complexity of public health interventions (Wight *et al*. 2016, Jones *et al*. 2017, Cambon *et al*. 2019). We considered any initiative or programme explicitly focusing on accommodating cultural groups’ values, beliefs, practices, behaviours, norms, or language. Cultural groups included Indigenous groups, tribal communities, and any form of group distinguished by its unique cultural traits. Studies were excluded if they did not describe a specific strategy (e.g. purely theoretical or problem-focused papers) or if they addressed maternal health without an intercultural component.
- **C (context):** Community, municipality, state, region, or country in LAC, defined as the region in the Americas that includes Mexico, South America, Central America, and the Caribbean. Studies conducted outside Latin America were excluded.

We included full-text primary or secondary research studies (qualitative, quantitative, or mixed-methods), considering both peer-reviewed and non-peer-reviewed articles, theses, and dissertations. We excluded abstracts, study protocols, books or book chapters, guides, and news articles. We considered studies published in English, Spanish, and Portuguese from 2013 onwards. We chose this timeframe because the most recent literature mapping on an overlapping topic covered publications from 1 January 1990 to 28 February 2013 (Coast *et al*. 2014).

### Search strategy and selection criteria

We searched nine databases: MEDLINE, Embase, Cochrane Library, Web of Science, ProQuest, APA PsycInfo, LILACS (Latin American and Caribbean Health Sciences Literature), SciELO (Scientific Electronic Library Online), and Redalyc. Grey literature was identified via Google Scholar and the official websites of the World Bank, World Health Organization (WHO), PAHO, and United Nations Children’s Fund (UNICEF). Additionally, we manually screened the reference lists of included reviews to identify further relevant sources. Database searches were conducted between March and June 2024, with grey literature searches and snowballing performed in October 2024. Detailed search strategies, including search dates and queries for each database, are provided in Supplementary File 3.

Given the interdisciplinary nature of the topic, spanning healthcare, anthropology, and the social sciences, we anticipated considerable variation in terminology and indexing. This was confirmed by reviewing a purposive sample of 10 known relevant studies and through exploratory hand searches. In some cases, studies may address intercultural aspects without using the term directly, instead referring to ‘vertical birth’, ‘traditional birth attendants’, or ‘culturally appropriate’. To account for this variation, we opted for a keyword-based search strategy and operationalized key concepts broadly.

We adopted UNESCO’s definition of interculturality, which refers to the equitable interaction among diverse cultures and the co-creation of shared cultural expressions through dialogue and mutual respect (UNESCO 2005). Considering the inconsistent use of this term in the literature, the search strategy also incorporated related concepts, including ‘culturally appropriate’, ‘culturally competent’, ‘culturally sensitive’, and ‘culturally responsive’ care.

The following working definitions were used for other key terms throughout the review: (i) TBAs are individuals who assist childbirth based on skills acquired through personal experience or apprenticeship (World Health Organization 1992); (ii) SBAs are trained and regulated professionals who provide evidence-based care to women and newborns during childbirth, including the management of or referral for complications (World Health Organization 2018); (iii) doulas are individuals with specialized training in labour support who are not part of the healthcare facility’s professional staff (World Health Organization 2016); and (iv) vertical birth refers to positions such as squatting, standing, sitting, hands-and-knees, and side-lying.

We developed and tested the initial search string for MEDLINE and then adapted it for the other databases. Using the Boolean operator AND, we combined three clusters of synonyms and related terms: (i) interculturality-related terms (e.g. ‘intercultural’, ‘culturally appropriate’, ‘traditional birth attendants’, ‘vertical birth’); (ii) maternal-neonatal health terms (e.g. ‘women’, ‘maternal’, ‘pregnancy’, ‘midwife’, ‘newborn’); and (iii) healthcare terms (e.g. ‘health’, ‘care’, ‘healthcare’). For Latin-American databases (LILACS, SciELO, and Redalyc), we also included terms in Spanish and Portuguese. Searches were limited to Title/Abstract fields and studies published from 2013 onwards.

### Study selection

The study selection process consisted of three phases. First, duplicate articles were removed using Zotero software. Next, titles and abstracts of all retrieved references were screened in Rayyan (Ouzzani *et al*. 2016) and classified as ‘potentially eligible’ or ‘excluded’. Finally, full texts of the ‘potentially eligible’ studies were reviewed to confirm eligibility or document reasons for exclusion. Two independent pairs of researchers (M.M.G.d.A. and R.I.R.C.; F.I.V. and T.B.d.N.F.) screened all records, with discrepancies resolved by a member of the other pair.

### Data extraction

We performed data extraction using a spreadsheet on Google Sheets. Two independent reviewers extracted data from the included studies, with any inconsistencies resolved by a third researcher. The following data were collected: first author’s surname, year of publication, journal, country, language, study design, terminology related to intercultural health, cultural groups addressed, and a description of the strategies.

### Methodological quality appraisal

Since this scoping review aims to map available evidence and identify strategies from descriptive and analytical studies, we did not apply any checklists or tools for assessing methodological quality, as recommended by the JBI Scoping Review Methodology Group (Peters *et al*. 2020b).

### Synthesis of results

The synthesis focused on identifying and reporting strategies to promote intercultural maternal and newborn care, as outlined in the literature and classified by the reviewing authors. A quantitative analysis, including descriptive statistics, was conducted using Microsoft Excel (2016), and tables were created to summarize the findings. We designed flowcharts using Figma software.

## Results

**Figure 1:**
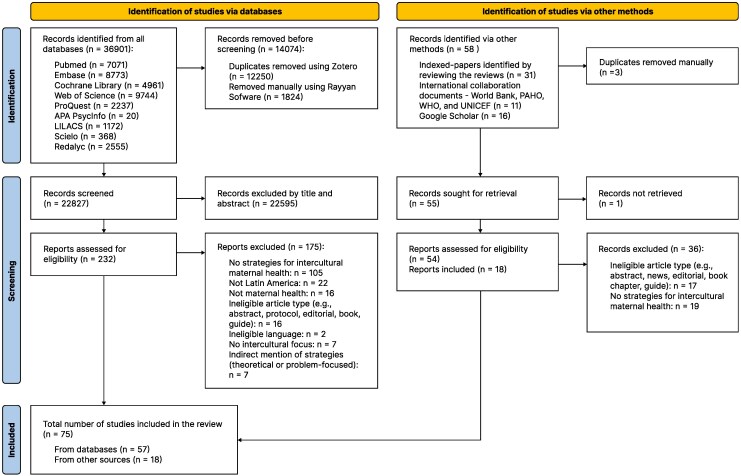
PRISMA flowchart.

Structured searches across nine databases yielded 36 901 references. After removing 14 074 duplicates, 22 827 references were screened based on titles and abstracts. Of these, 22 595 were excluded for not meeting the eligibility criteria. The remaining 232 studies were reviewed in full text, with 175 excluded and 57 meeting the inclusion criteria. Additional searches in other sources identified 58 references, of which 18 were included. In total, 75 studies were included in this review (Fig. 1).

### Study characteristics

Characteristics of each included study and the strategies reported are detailed in Supplementary File 4. Each strategy is characterized as proposed, partially implemented, or implemented. Most studies reported multiple and overlapping strategies, either in isolation or as part of a broader programme.

The LAC countries included in these studies were Argentina (*n* = 1), Bolivia (*n* = 5), Brazil (*n* = 3), Chile (*n* = 2), Colombia (*n* = 4), Ecuador (*n* = 8), Guatemala (*n* = 20), Haiti (*n* = 3), Mexico (*n* = 11), Nicaragua (*n* = 1), and Peru (*n* = 11). Additionally, six studies mentioned various countries, including Honduras, Cuba, Peru, Mexico, Colombia, Guatemala, Bolivia, Brazil, Argentina, Ecuador, and Nicaragua. Supplementary File 5 presents the geographical distribution of the studies.

Of the 75 articles, 56 (74.67%) were published in English, 15 (20%) in Spanish, and 4 (5.33%) in Portuguese. Concerning methodology, 48 studies (64%) employed qualitative methods, including action research, focus group discussions, structured and in-depth interviews, participatory research, programme descriptions, and ethnographic observation. Fifteen studies (20%) utilized quantitative or mixed methods, nine (12%) were review articles, and three (4%) were international collaboration reports from organizations such as the World Bank, PAHO, and UNICEF. Regarding publication platforms, 63 studies (84%) were published in peer-reviewed journals, one (1.33%) in a non-peer-reviewed journal, eight (10.67%) in institutional repositories for theses and dissertations, and three (4%) on the websites of international organizations, such as the World Bank, PAHO, and UNICEF.

In terms of cultural groups addressed, 56 studies (74.67%) concentrated on Indigenous populations; 12 (16%) addressed Indigenous peoples, Afro-descendants, Roma, and other ethnic groups in rural or urban settings; and 7 (9.33%) focused on different groups, including *quilombola* women (Freitas Júnior *et al*. 2018), rural communities, and Bolivian immigrants (Avellaneda Yajahuanca 2015). Regarding terminology, 50 studies (66.67%) used the terms ‘intercultural’ or ‘interculturality’, while 20 studies (26.67%) used alternative terms such as ‘culturally appropriate’, ‘culturally relevant’, ‘culturally adapted’, ‘culturally sensitive’, or ‘culturally competent’. Five studies (6.67%) did not use these terms but referenced related concepts, such as ‘traditional birth attendants’ or ‘traditional Mayan midwives’. Supplementary File 6 provides a graphical overview of these results, showing the number of publications by language, methods, platform, cultural group, terminology, and strategy.

### Strategies to promote intercultural maternal and newborn care

We identified 16 categories of strategies to promote intercultural maternal and newborn care: (i) promote intercultural childbirth practices; (ii) train SBAs and healthcare students in culturally sensitive approaches; (iii) provide culturally sensitive training for TBAs; (iv) culturally adapt birthing services; (v) empower, recognize, support, and integrate TBAs into the health system; (vi) ensure language accessibility in maternal health services and training; (vii) develop and implement intercultural health policies; (viii) facilitate dialogue to build a pluralistic health system; (ix) establish maternity houses; (x) deliver culturally relevant education for pregnant and postpartum women; (xi) incorporate new professionals into intercultural maternal care teams, such as intercultural health brokers; (xii) conduct research on intercultural maternal health; (xiii) build a diverse and culturally sensitive health workforce; (xiv) foster international cooperation; (xv) develop tools for monitoring and evaluation of intercultural initiatives; and (xvi) design culturally tailored social marketing campaigns to support maternal health. Most articles cited multiple strategies, with their distribution shown in Supplementary File 6.

**Figure 2:**
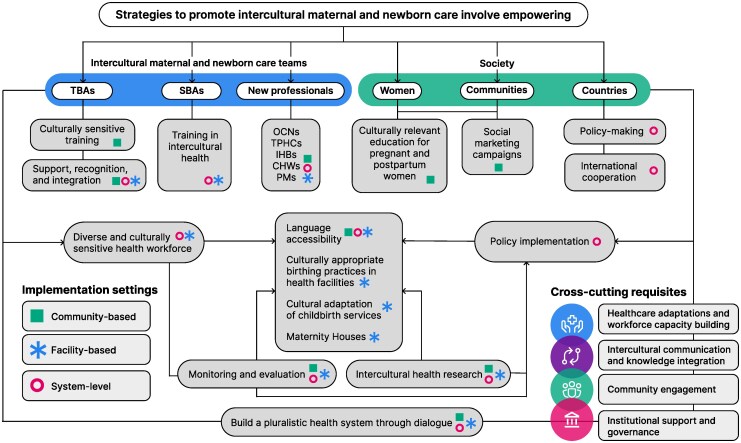
Intercultural maternal and newborn care framework. CHWs, community health workers; IHBs, intercultural health brokers; OCNs, obstetric care navigators; PMs, professional midwives; SBAs, skilled birth attendants; TBAs, traditional birth attendants; TPHCs, technicians in primary health care.

From the 16 categories, we identified four cross-cutting domains. These, observed across multiple strategies, included (i) healthcare adaptations and workforce capacity building, (ii) intercultural communication and knowledge exchange, (iii) community engagement, and (iv) institutional support and governance (Fig. 2). They were derived through an iterative interpretive process, examining recurring functions, target actors, and enabling conditions across the 16 categories and 75 included studies, and grouping conceptually related categories into broader domains.

Three levels of implementation (Fig. 2) also emerged inductively from this process, reflecting the primary operational contexts in which these strategies were delivered: community, facility, and health system levels. The facility level encompassed interventions implemented within health services and clinical settings, such as hospitals, health centres, and birthing facilities, involving SBAs. The community level referred to interventions operating outside formal health facilities, including those delivered in villages, homes, or community spaces, and involving families and community members. The health system level included measures addressing policy, governance, financing, workforce organization, or the structural reorganization of health services to support the integration of intercultural approaches.

Drawing on the 16 strategy categories, four cross-cutting domains, and the implementation contexts, we propose a framework for intercultural maternal and newborn care, presented in Fig. 2.

Table 1 presents the strategies, their descriptions, examples, and the studies that discuss them. Supplementary File 4 provides an overview of the strategies discussed in each study.

**Table 1:** Strategy categories, descriptions, and examples.

Author and year	Strategy	Description
26 studies Avellaneda Yajahuanca, 2015; Cañuta, 2017; Chomat *et al*., 2014; Coast *et al*., 2014; Coast *et al*., 2016; Cruz Llumiquinga, 2021; Del Mastro N. *et al*., 2021; Guerra-Reyes, 2016; Guerra-Reyes, 2019; Ibáñez-Cuevas *et al*., 2015; Lazo-Gonzales *et al*., 2023; Llamas and Mayhew, 2016; Llamas and Mayhew, 2018; Chia López and Díaz Herrera, 2019; Roosta-G., 2015; Vicente Martín, 2017; Matute *et al*., 2021; Miller and Smith, 2017; Moore, 2018; Morales, 2018; Sarmiento *et al*., 2024; Solís *et al*., 2023; Summer et al., 2019; Torri and Hollenberg, 2013; Trejos Serrato, 2022; UNICEF Argentina, 2023	Promote intercultural childbirth practices	Implement culturally appropriate birthing practices in health facilities. Examples: Authorize harmless traditional practices during labour, including certain medicines, ritual teas, herbal infusions, and traditional food consumption. Allow women to choose their preferred birthing position (e.g. vertical or squatting). Permit women to walk freely during labour. Allow women to wear traditional clothing or provide adequate clothes, such as a sterilized flannel gown. Allow the husband or another family member to remain in the room during labour and to cut the umbilical cord after delivery. Allow more than one person to accompany the mother during labour. Ensure an intercultural team, including TBAs and SBAs, assists births. Allow the family to take the placenta home for ritual burial. Maintain a warm environment during labour with electric heaters, soft massages, herbal infusions, and extra blankets. Minimize cervical dilation checks to reduce discomfort. Reduce exposure-related discomfort during labour. Permit traditional steam baths with medicinal herbs during labour.
19 studies Avellaneda Yajahuanca, 2015; Cañuta, 2017; Castillo-Santana *et al*., 2017; Chomat *et al*., 2014; Coast *et al*., 2014; Freitas Júnior *et al*., 2018; Guerra-Reyes, 2016; Guerra-Reyes, 2019; Hurtado Zambrana *et al*., 2014; Llamas and Mayhew, 2016; Llamas and Mayhew, 2018; Matute *et al*., 2021; Rangel Flores *et al*., 2017; Rojas, 2015; Sarmiento *et al*., 2022; Torri and Hollenberg, 2013; Torri, 2013; UNICEF Argentina, 2023; van Dijk et al., 2013	Train SBAs and healthcare students in culturally sensitive approaches	Train skilled health personnel (generally doctors, nurses, or professional midwives) in intercultural childbirth practices, cultural traditions, and values. Implement intercultural health approaches in the training of healthcare students.
19 studies Coast *et al*., 2014; Austad *et al*., 2020; Bautista-Valarezo *et al*., 2022; Blas *et al*., 2023; Chary *et al*., 2013; Cruz Llumiquinga, 2021; Del Mastro N. *et al*., 2021; Fahey *et al*., 2013; Garcia *et al*., 2018; Gusman *et al*., 2015; Hernandez *et al*., 2017; Hernandez *et al*., 2018; Kestler *et al*., 2020; Santana Mera and Boza, 2016; Sarmiento *et al*., 2024; Sousa and Corning-Davis, 2019; Stroux *et al*., 2016; Walton et al., 2016; World Bank Group, 2021	Provide culturally sensitive training for TBAs	Implement culturally adapted interventions co-developed with TBAs to improve referral practices, essential newborn care, and neonatal resuscitation and enhance recognition of obstetric emergencies. Examples: *Mamás del Río* Programme, PRONTO provider training, School of POWHER.
18 studies Avellaneda Yajahuanca, 2015; Coast *et al*., 2014; Cañuta, 2017; Chopel, 2014 ; Coast *et al*., 2016; Del Mastro N. *et al*., 2021; Gallegos *et al*., 2017; Guerra-Reyes, 2016; Guerra-Reyes, 2019; Roosta-G., 2015; Vicente Martín, 2017; Miller and Smith, 2017; Morales, 2018; Rojas, 2015; Solís *et al*., 2023; Torri and Hollenberg, 2013; Torri, 2013; Tovilla, 2022	Culturally adapt birthing services	Implement culturally appropriate services and facilities to create a home-like environment. Examples: Use low wooden beds, short stools, multicoloured bedsheets, heaters, and local textiles for decoration. Provide floor mats and birthing stools. Replace cold metal beds with wooden ones. Use darker-coloured bed sheets instead of white. Provide a small kitchen for heating water for herbal infusions. Use curtains and painted walls for a welcoming environment. Create a shared space for biomedical staff and TBAs to support patients, fostering the integration of traditional and western medicine. Create a herbal garden near the hospital to cultivate medicinal plants used in reproductive healthcare by TBAs.
17 studies Coast *et al*., 2014; Gallegos *et al*., 2017; Gusman *et al*., 2015; Hernandez *et al*., 2017; Hernandez *et al*., 2018; Kestler *et al*., 2020; Llamas and Mayhew, 2016; Miller and Smith, 2017; Nacht *et al*., 2022; Rojas, 2015; Santana Mera and Boza, 2016; Sarmiento *et al*., 2022; Suárez-Baquero and Champion, 2021; Summer et al., 2019; Torri and Hollenberg, 2013; Walsh et al., 2017; World Bank Group, 2021	Empower, recognize, support, and integrate TBAs into the health system	Fully integrate TBAs into the health systems. Examples: Incorporate TBAs into health services, particularly family health teams. Register active TBAs. Provide safe birthing kits. Offer material support to TBAs, such as stipends, to allow more time for patient care. Support apprenticeship programmes for TBAs. Recognize the invaluable work of TBAs. Establish associations for TBAs. Ensure formal documentation of births and maternal outcomes by TBAs. Empower TBAs to serve as trainers in educational programmes. Enable TBAs to lead educational campaigns within their communities.
14 studies Abrams *et al*., 2018; Chary *et al*., 2013; Chomat *et al*., 2014; Coast *et al*., 2016; Garcia *et al*., 2018; Hernandez *et al*., 2018; Ibáñez-Cuevas *et al*., 2015; Miller and Smith, 2017; Radoff *et al*., 2013; Sousa and Corning-Davis, 2019; Stroux *et al*., 2016; Torri and Hollenberg, 2013; UNICEF Argentina, 2023; van Dijk et al., 2013	Ensure language accessibility in maternal health services and training	Implement training programmes in participants’ native language, as these are more effective than translated programmes. Provide care for women in their native language. Increase the number of healthcare staff proficient in local languages.
14 studies Castillo-Santana *et al*., 2017; Coast *et al*., 2014; Del Mastro N. *et al*., 2021; Gallegos *et al*., 2017; Guerra-Reyes, 2016; Guerra-Reyes, 2019; Ibáñez-Cuevas *et al*., 2015; Llamas and Mayhew, 2016; Llamas and Mayhew, 2018; Chia López and Díaz Herrera, 2019; Vicente Martín, 2017; Moore, 2018; Olaza-Maguiña and De La Cruz-Ramirez, 2024; Rojas, 2015	Develop and implement intercultural health policies	Translate policy into practice by ensuring access to culturally relevant health services that comply with national regulations. Examples: Establish an Intercultural Health Office within the Ministry of Public Health. Develop and implement an intercultural birthing policy. Integrate vertical births into the healthcare system as part of an intercultural health policy. Promote a comprehensive Intercultural Health Policy. Provide financial incentives, such as cash allowances, to encourage adherence to maternal health policies. Scale successful policies, programmes, and initiatives by replicating validated local models nationwide.
11 studies Coast *et al*., 2014; Ibáñez-Cuevas *et al*., 2015; Guerra-Reyes, 2019; Lazo-Gonzales *et al*., 2023; Moore, 2018; Olivas *et al*., 2023; Penn-Kekana *et al*., 2017; Ruiz *et al*., 2013; Stollak *et al*., 2016; Tucker *et al*., 2013; van Braam *et al*., 2023	Establish maternity houses	Maternity houses include three approaches: Maternity waiting homes (MWHs): facilities near healthcare centres that accommodate pregnant women awaiting labour. MWHs aim to reduce barriers to accessing essential maternal and newborn care, ensuring timely interventions for childbirth and complications. Casas maternas (community birthing centres): facilities near health centres or hospitals that provide culturally appropriate maternal care. These centres allow women to give birth in their preferred position, attended by traditional birth attendants (TBAs) and supported by family and cultural rituals. Maternity houses for high-risk pregnancies: facilities dedicated to managing high-risk pregnancies referred from remote areas, offering specialized care and support.
10 studies Abrams *et al*., 2018; Cardona-Arias *et al*., 2015; Castillo-Santana *et al*., 2017; Chomat *et al*., 2014; Gallegos *et al*., 2017; Pulido Hernández, 2022; Rangel Flores *et al*., 2017; Solís *et al*., 2023; Torri, 2013; UNICEF Argentina, 2023	Facilitate dialogue to build a pluralistic health system	Establish dialogue platforms mediated by Indigenous and western health authorities to foster mutual recognition and understanding of each health system's principles. Promote cultural exchange, awareness, and respectful dialogue. Examples: Engage native advisers in planning to ensure cultural relevance. Facilitate shared decision-making between professionals in western and traditional medicine. Encourage knowledge exchange on Indigenous and western medical practices. Incorporate the perspectives, needs, and priorities of chiefs and community leaders in intercultural maternal health planning.
10 studies Abrams *et al.*, 2018; Atyeo *et al.*, 2017; Blas *et al.*, 2023; Coast *et al.*, 2014; Del Mastro N *et al.*, 2021; Ortiz Anaya and Guillermo Rojas, 2022; Radoff *et al.*, 2013; UNICEF Argentina, 2023; Walsh *et al.*, 2017; World Bank Group, 2021	Deliver culturally relevant education for pregnant and postpartum women	Incorporate cultural beliefs into health education programmes. Examples: Integrate cultural perspectives into group prenatal care, breastfeeding education, and newborn care to improve community engagement and understanding. Adapt the Kangaroo-Mother method for home use, ensuring it aligns with cultural practices and supports caregivers in providing effective newborn care. Utilize culturally appropriate, community-based radio soap operas to enhance women’s awareness of pregnancy danger signs, tailoring the content to be culturally relevant and accessible.
8 studies Austad *et al.*, 2020; Gallegos *et al.*, 2017; Pulido Hernández, 2022; Sarmiento *et al.*, 2022; Sarmiento *et al.*, 2024; Summer *et al.*, 2017; Summer and Walker, 2018; Summer, Walker and Guendelman *et al.*, 2019	Incorporate new professionals into intercultural maternal care teams	Integrate new professionals into intercultural maternal care teams. Examples: Obstetric care navigators (OCNs): trained Maya women who act as OCNs, facilitating referrals from TBAs to public hospitals. Technicians in primary healthcare: individuals with 2 years of training serving as health promoters and intermediaries between public health professionals and the local population. In predominantly Indigenous areas, they speak the local language and understand cultural health beliefs and practices. Intercultural health brokers: bilingual community members trained as health brokers who support traditional midwives and improve collaboration with western healthcare providers. Professional midwives: reintroduce professional midwives into the health system to increase skilled birth attendants in rural and Indigenous communities. Establish a government-accredited midwifery school to train skilled birth attendants in rural and Indigenous areas. Applicants must be Indigenous women who speak the local language. Provide training for professional midwives to work alongside TBAs.
6 studies Del Mastro N. *et al.*, 2021; Roosta-G., 2015; Sarmiento *et al.*, 2020a; Sarmiento *et al.*, 2020b; Sarmiento *et al.*, 2021; Torri, 2013	Conduct research on intercultural maternal health	Conduct formative research to develop programmes. Examples: Generate knowledge about medicinal plants. Use participatory research and intercultural dialogue to improve maternal health. Map Indigenous knowledge to incorporate their voices into research and decision-making in maternal health. Intercultural researchers can facilitate intercultural dialogue in maternal health through literature reviews and stakeholder mapping.
4 studies Chomat *et al.*, 2014; Coast *et al.*, 2016; Guerra-Reyes, 2016; Miller and Smith, 2017	Build a diverse and culturally sensitive health workforce	Employ a more diverse and culturally sensitive health workforce. Increase the number of healthcare staff sensitive to Indigenous norms and practices.
4 studies Castillo-Santana *et al.*, 2017; Pan American Health Organization, 2023; UNICEF Argentina, 2023; World Bank Group, 2021	Foster international cooperation	Facilitate experience exchange in intercultural childbirth care with other countries.
4 studies Hurtado Zambrana *et al.*, 2014; Pan American Health Organization, 2023; Samuel, 2016; Stollak *et al.*, 2016	Develop tools for monitoring and evaluation of intercultural initiatives	Examples: Implement tools to assess the adoption of an intercultural approach in maternal health services. Implement a citizen monitoring initiative to ensure healthcare services adhere to the intercultural birthing policy. Promote continuous training and quality assessment of the work in maternity houses by professionals like obstetric nurses. Promote public discussions on intercultural health to raise awareness and engagement.
4 studies Fahey *et al.*, 2013; Kestler *et al.*, 2020; Radoff *et al.*, 2013; Ruiz *et al.*, 2013	Design culturally tailored social marketing campaigns	Examples: Promote social marketing campaigns to improve the perception of the quality of care in community clinics. Promote marketing campaigns to raise awareness about danger signs and encourage pregnant women to give birth at the nearest healthcare facility. Promote radio campaigns encouraging facility-based births. Develop communication strategies to increase awareness of maternity houses.

TBAs, traditional birth attendants; SBAs, skilled birth attendants; OCNs, obstetric care navigators.

## Discussion

This scoping review identified 75 studies on strategies to promote intercultural maternal and newborn care in LAC, covering 16 approaches across 13 countries. Guatemala, Mexico, and Peru had the highest representation. Most studies were published in peer-reviewed journals, in English, and focused on Indigenous populations, with the majority using the terms ‘intercultural’ or ‘interculturality’. The study design was qualitative for most studies, with a predominance of ethnographic research, reflecting the established role of ethnography in global health (MacDonald 2017).

The 16 strategy categories identified in this review are situated within four cross-cutting domains that span facility-based, community-based, and system-level settings: (i) healthcare adaptations and workforce capacity building, (ii) intercultural communication and knowledge exchange, (iii) community engagement, and (iv) institutional support and governance. Based on these findings, we introduce a novel framework for intercultural maternal and newborn care. For example, promoting intercultural childbirth practices (Table 1) is a form of healthcare adaptation that requires a trained and culturally competent health workforce (i). It also depends on effective intercultural communication and the creation of shared knowledge spaces (ii), developed in collaboration with women, families, TBAs, and communities (iii). The implementation and sustainability of these strategies require institutional support and mechanisms for monitoring and evaluation (iv), with ongoing community involvement (iii).

Within the domain ‘healthcare adaptations and workforce capacity’, our findings align with a systematic mapping conducted by Coast and colleagues (Coast *et al*. 2014), who reviewed interventions addressing cultural factors influencing women’s use of skilled maternity care between 1990 and 2013. They identified strategies such as allowing family and TBA participation during childbirth, providing benches for vertical delivery, and establishing maternity waiting homes (MWHs). These interventions primarily targeted Indigenous communities in LAC and Australia. Empirical studies from Eritrea (Andemichael *et al*. 2009, Bekele and Umubyeyi 2018, Selbana *et al*. 2020) and Ethiopia also reported MWHs as effective in increasing skilled deliveries. Regarding workforce capacity, Coast and colleagues (Coast *et al*. 2014) highlighted efforts across countries, such as the USA, Canada, Nepal, Pakistan, and China, to diversify health staff and train SBAs in culturally appropriate care. Similar to the ‘intercultural health brokers’ identified in our review, they described ‘cultural brokers’ or ‘linkworkers’ who support immigrant and ethnic minority groups in the USA and UK by bridging gaps between service users and providers.

TBAs are another key stakeholder in intercultural maternal care. Although untrained in the standards of formal healthcare systems, TBAs deeply understand their communities’ struggles, customs, and traditions, while also being highly respected and trusted in many cultures (Kassie *et al*. 2022). With appropriate training, equipment, support, and integration into the healthcare systems, they become pivotal stakeholders in improving maternal and postpartum care, reducing perinatal, neonatal, and maternal mortality (Wilson *et al*. 2011), and increasing the rate of skilled birth attendance (Vieira *et al*. 2012). We identified two main strategies involving TBAs: (i) culturally sensitive training and (ii) empowering, supporting, and integrating TBAs into healthcare systems. These findings are consistent with studies from Africa (Kassie *et al*. 2022, Musie *et al*. 2022) and Asia (Shaikh *et al*. 2014) and review studies from the Americas (Sarmiento *et al*. 2021) and other regions (Miller and Smith 2017, Rutledge *et al*. 2024). Notably, we observed three distinct approaches to these strategies: (i) inclusion focused on TBA participation during childbirth, assigning new roles or modifying their practices to support western care; (ii) support for TBAs on their own terms (Sarmiento *et al*. 2022), through fair remuneration, provision of supplies, culturally appropriate training, and formal integration into health systems (Miller and Smith 2017, Garcia *et al*. 2018); and (iii) concerns raised, particularly in studies focused on Indigenous populations, that training TBAs according to western standards, even when culturally adapted, may devalue the Indigenous cosmovisions (Sarmiento *et al*. 2021) and reinforce traditional systems’ subordination.

These differing perspectives reveal the tensions between Indigenous knowledge systems and western healthcare, as well as varied interpretations of interculturality. Many studies identified as intercultural those strategies that centred on modifying western health services to incorporate traditional practices and foster dialogue. This is the predominant perspective in the global literature on intercultural maternal care. However, in LAC, it is also discussed that restricting the intercultural encounter to ‘dialogue’ can obscure deeper power imbalances that marginalize Indigenous peoples (Tubino 2004). In this context, two distinct approaches to interculturality emerged in our review: functional and critical, as described by Tubino (2004). Functional interculturality acknowledges cultural differences but does not challenge the underlying structural inequalities within health systems (Tubino 2004). In contrast, critical interculturality calls for transforming these power structures to establish equitable relationships between the State and Indigenous peoples, seeking fundamental changes in how policies and services are designed and delivered (Tubino 2002, 2004).

Although most included studies (74.67%) focused on Indigenous groups in countries with large Indigenous populations, functional interculturality was the dominant pattern in the strategies. Systemic, institutional, economic, and geographic barriers were frequently acknowledged and discussed, but efforts to address them directly were limited. This may help explain why, despite the existence of intercultural health policies in LAC, countries continue to face mistrust between western health services and Indigenous traditional care (Sarmiento *et al*. 2021), contributing to poor maternal outcomes (Paulino *et al*. 2019, Sáenz *et al*. 2024). Common strategies, such as vertical birthing rooms (Llamas and Mayhew 2016, Matute *et al*. 2021) and TBA training programmes (Gusman *et al*. 2015, Garcia *et al*. 2018), are relatively low-cost and politically visible, which facilitates adoption and alignment with national policies. However, when these efforts are isolated or lack sustained support, such as vertical birthing rooms without continued TBA involvement, their impact on trust and service utilization remains modest (Llamas and Mayhew 2016, Gallegos *et al*. 2017). Therefore, symbolic inclusion, in the absence of monitoring, evaluation, and community engagement, reinforces persistent implementation gaps. Structural barriers, including underfunded health systems, power asymmetries, and lack of formal recognition for Indigenous practices, further influence the delivery of intercultural services (Avellaneda Yajahuanca 2015, Guerra-Reyes 2016, 2019, Hernandez *et al*. 2018, Llamas and Mayhew 2018, Blas *et al*. 2023). Together, these challenges highlight the limitations of functional interculturality and reinforce the need to advance towards critical interculturality.

Achieving this shift in maternal-neonatal health requires progressing beyond theoretical interculturality towards active transformation of healthcare systems. In our scoping review, strategies with strong institutional support and community engagement (cross-cutting domains iii and iv) were more successful in improving maternal and newborn outcomes. For example, Ecuador’s intercultural policy at San Luis de Otavalo Hospital, which integrates Indigenous practices and involves sustained training of hospital staff, was recognized by the WHO for reducing maternal mortality among Kichwa and mestizo women (Matute *et al*. 2021). In rural Amazon, the Mamas del Río programme improved essential newborn care indicators in both home and institutional births by combining community empowerment with the strengthening of local health facilities (Del Mastro *et al*. 2021, Blas *et al*. 2023). In rural Guatemala, an accompaniment model in which Mayan women trained as obstetric care navigators collaborated with TBAs enhanced system navigation and maternal health service utilization (Austad *et al*. 2020).

Additionally, culturally appropriate TBA training can improve their ability to recognize obstetric and neonatal emergencies and make timely referrals (Fahey *et al*. 2013, Stroux *et al*. 2016, Walton *et al*. 2016, Hernandez *et al*. 2018, Kestler *et al*. 2020). These training approaches integrate TBA roles and customs into simulations and hands-on scenarios, adapt to literacy and language, co-develop educational materials, and employ low-cost technology to support learning (Fahey *et al*. 2013, Walton *et al*. 2016, Garcia *et al*. 2018, Hernandez *et al*. 2018, Austad *et al*. 2020, Kestler *et al*. 2020, Blas *et al*. 2023).

Although these strategies have shown promise, their impact on maternal and perinatal mortality remains unclear due to the limited quantitative data. As noted by Austad *et al*. (2020), randomized trials adequately powered to detect differences in maternal and neonatal outcomes, alongside systematic assessments of patient satisfaction, would provide valuable evidence. Another gap concerns domain (ii), intercultural communication and knowledge exchange. While most studies highlight the role of intercultural competence in facilitating two-way learning between healthcare providers, patients, and their communities, clear mechanisms to promote this exchange and mitigate potential tensions are not well defined. The majority of strategies tend to target specific groups, such as TBAs or SBAs, rather than the development of intercultural health teams. These represent important directions for future research. Interestingly, research itself has emerged as a strategy to promote intercultural maternal and newborn care (Table 1).

### Limitations and strengths

This study has several limitations. First, despite a comprehensive search, some studies, especially theses in institutional repositories, may have been missed. The exclusion of books, chapters, and conference proceedings could have further limited the scope of evidence captured. Another potentially relevant source would be the CINAHL database, which was not included due to a lack of access at the time of protocol registration. However, searches in nine databases, grey literature, and hand searches likely mitigated these limitations. This process allowed the identification of theses and dissertations from LAC universities and materials from the websites of international organizations, which contributed to the body of evidence on this topic.

The time elapsed between the search and manuscript submission represents an additional limitation. However, the commonly used 12-month threshold for updating searches has been challenged as context-dependent. Large, complex, and predominantly qualitative evidence syntheses, such as this scoping review, are generally considered less susceptible to becoming outdated than intervention effectiveness reviews in rapidly evolving fields (Stokes *et al*. 2023, Alexander *et al*. 2024), as the inclusion of more recent studies is less likely to materially change the overall interpretation of the mapped evidence. As such, this scoping review provides a useful foundation for future research.

Second, the lack of standardized terminology for ‘interculturality’ and ‘traditional birth attendants’ posed challenges in study identification. While many studies employed terms such as ‘intercultural’ or ‘interculturality’, others explored related topics without explicitly using these terms. Similarly, terminology for TBAs is highly variable (e.g. *comadronas*, *matwon*, traditional midwives, Mayan midwives, *parteras tradicionales*). This diversity in terminology was reflected in the search results, as some relevant articles were not retrieved by database searches but were identified through hand searching. Greater consistency in terminology would strengthen comparability across studies and facilitate future evidence synthesis, representing an important methodological consideration in this field.

Despite these limitations, our study has several strengths. The research team was multidisciplinary, with members from three Latin American countries, combining experience in primary care, social sciences, policy, and ethnographic research involving Indigenous women and TBAs. This diversity strengthened the contextual interpretation of the findings. In addition, to our knowledge, this was the first scoping review dedicated to intercultural maternal and newborn care. The search was comprehensive, and JBI and PRISMA guidelines were followed. This study introduces a novel framework for intercultural maternal and newborn care, which can inform future efforts in this field.

### Reflections

Analysing the strategies that emerged from this scoping review, a notable characteristic was their multi-level nature, particularly in Indigenous communities. Implementing and translating them into health system improvements requires considering the interplay between individual and social environmental factors. Mapping the strategies against a Social Ecological Model (SEM) for health promotion, the 16 categories crossed all the levels described by McLeroy *et al*. (1988): (i) individual, (ii) interpersonal, (iii) institutional or organizational, (iv) community, and (v) public policy. Our proposed framework is grounded in the SEM, recognizing that changes in social environments shape individual behaviours, while individual and community engagement drive the structural transformations necessary for advancing intercultural care.

Within the individual and interpersonal levels, there are strategies targeting one or more of the interconnected stakeholders involved in maternal and newborn care, including women, family members, TBAs, SBAs, community leaders, policymakers, researchers, and citizens. Each stakeholder has different perceptions about the barriers and facilitators for promoting intercultural care (Tucker *et al*. 2013, van Dijk *et al*. 2013, Summer *et al*. 2017, Llamas and Mayhew 2018). Critical interculturality can offer a pathway to address those discrepancies, reduce tensions between western and traditional medicine, align expectations, foster equal and reciprocal knowledge sharing, and sensitize health workers to the value of TBAs as agents of change. However, strategies to address critical interculturality are less consistently documented across the included studies, representing an important gap for future research and policy attention. Although several initiatives aimed to reach institutional, community, and policy levels, their impact remained limited, reflecting the persistence of structural barriers.

Beyond the LAC context, these findings may also inform strategies in other LMICs facing similar structural barriers and tensions between biomedical and traditional healthcare systems. In particular, we highlight the fundamental role of TBAs’ knowledge, voices, and experiences (Kruske and Barclay 2004). Their formal recognition within health systems, inclusion in training programmes, initiative co-design, and community liaison roles are important steps for effective implementation. LAC countries may further benefit from structured mechanisms, such as participatory audits and intercultural health committees, to address persistent discrepancies between policy and healthcare practice (Gallegos *et al*. 2017). The application of monitoring and evaluation tools can support citizens and stakeholders in advocating for improvements in intercultural healthcare. At the individual and interpersonal levels, preparing healthcare teams to provide person-centred care is essential. Intercultural care requires recognizing cultural dynamism and intra-community diversity, as not all individuals within a group share the same values or care preferences.

Our proposed framework integrates these complex processes into an actionable model for improving intercultural maternal and newborn care. It shares common ground with existing frameworks for health system strengthening and health promotion while also addressing specific dimensions in this context of care. The WHO health systems framework (World Health Organization 2007) describes six building blocks: leadership and governance, service delivery, health system financing, health workforce, medical products, vaccines and technologies, and health information systems. Complementarily, the Ottawa Charter for Health Promotion (World Health Organization 1986) reinforces that effective implementation requires simultaneous action across five areas: building healthy public policy, creating supportive environments, strengthening community action, developing personal skills, and reorienting health services. Our proposed framework acknowledges the interdependence of these elements while connecting intercultural care across actors, settings, and domains. It centres cultural dialogue, community agency, and the role of traditional knowledge holders as active participants in maternal and newborn care. In doing so, it demonstrates how intercultural dimensions can be operationalized within health system strengthening and health promotion efforts more broadly.

Reorienting health systems to intercultural approaches has the potential to strengthen trust, improve access, and promote more responsive maternal health services. It may also contribute to the overarching goals of equity, quality, and universal health coverage.

## Conclusion

Strategies to promote intercultural maternal and newborn care in LAC are multifaceted, involving diverse stakeholders and intersecting fields such as healthcare, policy, and social sciences. Although these approaches can improve maternal-neonatal outcomes and advance health equity, structural barriers challenge their implementation. Addressing these limitations requires strengthening culturally appropriate health services, building workforce capacity, fostering intercultural communication and knowledge exchange, supporting community participation, and ensuring ongoing institutional and policy support. The proposed framework offers a foundation to guide future research and practice towards a more equitable model of maternal healthcare within LAC and, potentially, in other global contexts.

## Supplementary Material

daag082_Supplementary_Data

## Data Availability

The data underlying this article are available in the article and in its online supplementary material.

## References

[daag082-B1] Abrams JA, Forte J, Bettler C et al Considerations for implementing group-level prenatal health interventions in low-resource communities: lessons learned from Haiti. J Midwifery women’s Health 2018;63:121–6. 10.1111/jmwh.12684 29359879

[daag082-B2] Alexander L, Cooper K, Peters MD et al Large scoping reviews: managing volume and potential chaos in a pool of evidence sources. J Clin Epidemiol 2024;170:111343. 10.1016/j.jclinepi.2024.111343 38582403

[daag082-B3] Al-Mubarak A, Ahilan B, Dasgupta T et al The impact of culture on access to and utilisation of maternity care amongst muslim women in high-income countries: a qualitative systematic review. BJOG 2025;132:1996–2008. 10.1111/1471-0528.18290 40693305 PMC12592763

[daag082-B4] Anand R, Lahiri I. Intercultural competence in health care: developing skills for interculturally competent care. In: The SAGE Handbook of Intercultural Competence. Thousand Oaks, CA: SAGE Publications, 2009, 387–402.

[daag082-B5] Andemichael G, Haile B, Kosia A et al Maternity waiting homes: a panacea for maternal/neonatal conundrums in Eritrea. J Eritrean Med Assoc 2009;4:18–21. 10.4314/jema.v4i1.52112

[daag082-B6] Arksey H, O’Malley L. Scoping studies: towards a methodological framework. Int J Soc Res Methodol 2005;8:19–32. 10.1080/1364557032000119616

[daag082-B7] Armstrong R, Hall BJ, Doyle J et al Scoping the scope’ of a cochrane review. J Public Health 2011;33:147–50. 10.1093/pubmed/fdr015 21345890

[daag082-B8] Atyeo NN, Frank TD, Vail EF et al Early initiation of breastfeeding among Maya mothers in the western highlands of Guatemala: practices and beliefs. J Hum Lact 2017;33:781–9. 10.1177/0890334416682729 28107098

[daag082-B9] Aubel J, Chibanda D. The neglect of culture in global health research and practice. BMJ Glob Health 2022;7:e009914. 10.1136/bmjgh-2022-009914

[daag082-B10] Austad K, Juarez M, Shryer H et al Obstetric care navigation: results of a quality improvement project to provide accompaniment to women for facility-based maternity care in rural Guatemala. BMJ Qual Saf 2020;29:169–78. 10.1136/bmjqs-2019-009524 PMC7045784 31678958

[daag082-B11] Avellaneda Yajahuanca RS . A experiência de gravidez, parto e pós-parto das imigrantes bolivianas e seus desencontros na cidade de São Paulo - Brasil. Doctoral thesis. Faculdade de Saúde Pública, Universidade de São Paulo, São Paulo, 2015.

[daag082-B12] Bautista-Valarezo E, Espinosa ME, Michels NRM et al Culturally adapted flowcharts in obstetric emergencies: a participatory action research study. BMC Pregnancy Childbirth 2022;22:772. 10.1186/s12884-022-05105-z 36229785 PMC9564086

[daag082-B13] Bekele BB, Umubyeyi A. Maternity waiting homes and skilled delivery in Ethiopia: review of strategy and implementation to drive sustainable development goals. Med Pract Rev 2018;9:19–26. 10.5897/MPR2018.0137

[daag082-B14] Blas MM, Reinders S, Alva A et al Effect of the Mamás del Río programme on essential newborn care: a three-year before-and-after outcome evaluation of a community-based, maternal and neonatal health intervention in the Peruvian Amazon. Lancet Reg Health Am 2023;28:100634. 10.1016/j.lana.2023.100634 38076412 PMC10701122

[daag082-B15] Byrne A, Morgan A. How the integration of traditional birth attendants with formal health systems can increase skilled birth attendance. Int J Gynaecol Obstet 2011;115:127–34. 10.1016/j.ijgo.2011.06.019 21924419

[daag082-B16] Cambon L, Terral P, Alla F. From intervention to interventional system: towards greater theorization in population health intervention research. BMC Public Health 2019;19:339. 10.1186/s12889-019-6663-y 30909891 PMC6434858

[daag082-B17] Cañuta CLFA . Las múltiples paradojas de la política de salud intercultural en la atención de salud materna que promueve el estado chileno. El caso del Hospital Kalvu Llanka de Cañete. Master's thesis. Centro de Investigaciones y Estudios Superiores en Antropología Social, 2017.

[daag082-B18] Cardona-Arias JA, Rivera-Palomino Y, Carmona-Fonseca J. Expresión de la interculturalidad en salud en un pueblo emberá-chamí de Colombia. Revista Cubana de Salud Pública 2015; 41:77–93. https://www.redalyc.org/articulo.oa?id=21438819008

[daag082-B19] Castillo-Santana PT, Vallejo-Rodríguez ED, Cotes-Cantillo KP et al Salud materna indígena en mujeres Nasa y Misak del Cauca, Colombia: tensiones, subordinación y diálogo intercultural entre dos sistemas médicos. Saúde e Sociedade 2017;26:61–74. 10.1590/s0104-12902017168743

[daag082-B20] Chary A, Díaz AK, Henderson B et al The changing role of indigenous lay midwives in Guatemala: new frameworks for analysis. Midwifery 2013;29:852–8. 10.1016/j.midw.2012.08.011 23410502

[daag082-B21] Chia López SI, Díaz Herrera A. Implementación de la política sectorial de salud intercultural, relacionada a la salud materna neonatal, en mujeres quechuas de 15 a 49 años, del distrito de Vilcashuamán y Saurama, provincia de Vilcashuamán, departamento de Ayacucho 2018. Master's thesis. Pontificia Universidad Católica del Perú, Lima, 2019.

[daag082-B22] Chomat AM, Solomons NW, Montenegro G et al Maternal health and health-seeking behaviors among indigenous Mam mothers from Quetzaltenango, Guatemala. Rev Panam Salud Publica 2014;35:113–20. https://pubmed.ncbi.nlm.nih.gov/24781092/24781092

[daag082-B23] Chopel AM . Reproductive health in indigenous Chihuahua: giving birth ‘alone like the goat’. Ethn Health 2014;19:270–96. 10.1080/13557858.2013.771150 23444879

[daag082-B24] Coast E, Jones E, Lattof SR et al Effectiveness of interventions to provide culturally appropriate maternity care in increasing uptake of skilled maternity care: a systematic review. Health Policy Plan 2016;31:1479–91. 10.1093/heapol/czw065 27190222 PMC5091340

[daag082-B25] Coast E, Jones E, Portela A et al Maternity care services and culture: a systematic global mapping of interventions. PLoS One 2014;9:e108130. 10.1371/journal.pone.0108130 25268940 PMC4182435

[daag082-B26] Cruz Llumiquinga FE . Impacto de la salud materna con enfoque intercultural en el desarrollo humano de mujeres indígenas de la provincia de Imbabura entre el año 2008 a 2018. Master’s thesis. FLACSO. Sede Académica Argentina, Buenos Aires, 2021.

[daag082-B27] Degrie L, Gastmans C, Mahieu L et al How do ethnic minority patients experience the intercultural care encounter in hospitals? A systematic review of qualitative research. BMC Med Ethics 2017;18:2. 10.1186/s12910-016-0163-8 28103849 PMC5244561

[daag082-B28] Del Mastro NI, Tejada-Llacsa PJ, Reinders S et al Home birth preference, childbirth, and newborn care practices in rural Peruvian Amazon. PLoS One 2021;16:e0250702. 10.1371/journal.pone.0250702 33945560 PMC8096074

[daag082-B29] Fahey JO, Cohen SR, Holme F et al Promoting cultural humility during labor and birth: putting theory into action during PRONTO obstetric and neonatal emergency training. J Perinat Neonatal Nurs 2013;27:36–42. 10.1097/JPN.0b013e31827e478d 23360940

[daag082-B30] Freitas Júnior RAdeO, Santos CAD, Lisboa LL et al Incorporando a Competência Cultural para Atenção à Saúde Materna em População Quilombola na Educação das Profissões da Saúde. Revista Brasileira de Educação Médica 2018;42:100–9. 10.1590/1981-52712015v42n2rb20170086

[daag082-B31] Gallegos CA, Waters WF, Kuhlmann AS. Discourse versus practice: are traditional practices and beliefs in pregnancy and childbirth included or excluded in the Ecuadorian health care system? Int Health 2017;9:105–11. 10.1093/inthealth/ihw053 27993953

[daag082-B32] Gamlin J, Holmes S. Preventable perinatal deaths in indigenous Wixárika communities: an ethnographic study of pregnancy, childbirth and structural violence. BMC Pregnancy Childbirth 2018;18:243. 10.1186/s12884-018-1870-6 29914405 PMC6006582

[daag082-B33] Garcia K, Dowling D, Mettler G. Teaching Guatemalan traditional birth attendants about obstetrical emergencies. Midwifery 2018;61:36–8. 10.1016/j.midw.2018.02.012 29524774

[daag082-B34] Guerra-Reyes L . Implementing a culturally appropriate birthing policy: ethnographic analysis of the experiences of skilled birth attendants in Peru. J Public Health Policy 2016;37:353–68. 10.1057/jphp.2016.19 27193501

[daag082-B35] Guerra-Reyes L . Numbers that matter: right to health and Peruvian maternal strategies. Med Anthropol 2019;38:478–92. 10.1080/01459740.2018.1563080 30657710

[daag082-B36] Gusman CR, Viana AP de AL, Miranda MAB et al [Inclusion of traditional birth attendants in the public health care system in Brazil: reflecting on challenges]. Rev Panam Salud Publica 2015;37:365–70. https://pubmed.ncbi.nlm.nih.gov/26208209/26208209

[daag082-B37] Hernandez S, Oliveira J, Jones L et al Impact of standardized prenatal clinical training for traditional birth attendants in rural Guatemala. Healthcare (Basel, Switzerland) 2018;6:60. 10.3390/healthcare6020060 29890732 PMC6023520

[daag082-B38] Hernandez S, Oliveira JB, Shirazian T. How a training program is transforming the role of traditional birth attendants from cultural practitioners to unique health-care providers: a community case study in rural Guatemala. Front Public Health 2017;5:111. 10.3389/fpubh.2017.00111 28580354 PMC5437202

[daag082-B39] Hurtado Zambrana JL, Manjón Calvimontes N, Pérez Mendoza R et al Factores relacionados a la atención con enfoque intercultural en los servicios de salud materna Sucre, 2009. ENFERvida 2014;2:8–16. https://revistas.usfx.bo/index.php/enfervida/article/view/38

[daag082-B40] Ibáñez-Cuevas M, Heredia-Pi IB, Meneses-Navarro S et al Labor and delivery service use: indigenous women’s preference and the health sector response in the Chiapas Highlands of Mexico. Int J Equity Health 2015;14:156. 10.1186/s12939-015-0289-1 26698570 PMC4688940

[daag082-B41] Jones E, Lattof SR, Coast E. Interventions to provide culturally-appropriate maternity care services: factors affecting implementation. BMC Pregnancy Childbirth 2017;17:267. 10.1186/s12884-017-1449-7 28854901 PMC5577805

[daag082-B42] Kassie A, Wale A, Girma D et al The role of traditional birth attendants and problem of integration with health facilities in remote rural community of West Omo Zone 2021: exploratory qualitative study. BMC Pregnancy Childbirth 2022;22:425. 10.1186/s12884-022-04753-5 35596165 PMC9123652

[daag082-B43] Kestler E, Ambrosio G, Hemming K et al An integrated approach to improve maternal and perinatal outcomes in rural Guatemala: a stepped-wedge cluster randomized trial. Int J Gynaecol Obstet 2020;151:109–16. 10.1002/ijgo.13262 32524605

[daag082-B44] Kruske S, Barclay L. Effect of shifting policies on traditional birth attendant training. J Midwifery Women’s Health 2004;49:306–11. 10.1016/j.jmwh.2004.01.005 15236710

[daag082-B45] Lazo-Gonzales AO, Sarmiento-Casavilca T, Espinosa-Henao OE et al Looking at maternal health of Asháninka communities from the conceptual framework of the accessibility of care. Int J Equity Health 2023;22:154. 10.1186/s12939-023-01943-1 37580769 PMC10426136

[daag082-B46] Leininger M . Culture care theory, research, and practice. Nurs Sci Q 1996;9:71–8. 10.1177/089431849600900208 8710313

[daag082-B47] Levac D, Colquhoun H, O’Brien KK. Scoping studies: advancing the methodology. Implement Sci 2010;5:69. 10.1186/1748-5908-5-69 20854677 PMC2954944

[daag082-B48] Llamas A, Mayhew S. The emergence of the vertical birth in Ecuador: an analysis of agenda setting and policy windows for intercultural health. Health Policy Plan 2016;31:683–90. 10.1093/heapol/czv118 26758539 PMC4916315

[daag082-B49] Llamas A, Mayhew S. Five hundred years of medicine gone to waste’? Negotiating the implementation of an intercultural health policy in the Ecuadorian Andes. BMC Public Health 2018;18:686. 10.1186/s12889-018-5601-8 29866186 PMC5987654

[daag082-B50] MacDonald M . Why ethnography matters in global health: the case of the traditional birth attendant. J Glob Health 2017;7:020302. 10.7189/jogh.07.020302

[daag082-B51] Matute SED, Martinez EZ, Donadi EA. Intercultural childbirth: impact on the maternal health of the Ecuadorian Kichwa and mestizo people of the Otavalo region. Rev Bras Ginecol Obstet 2021;43:14–9. 10.1055/s-0040-1721353 33513631 PMC10183941

[daag082-B52] McLeroy KR, Bibeau D, Steckler A et al An ecological perspective on health promotion programs. Health Educ Q 1988;15:351–77. 10.1177/109019818801500401 3068205

[daag082-B53] Miller T, Smith H. Establishing partnership with traditional birth attendants for improved maternal and newborn health: a review of factors influencing implementation. BMC Pregnancy Childbirth 2017;17:365. 10.1186/s12884-017-1534-y 29052533 PMC5649078

[daag082-B54] Moore M-A . Interculturality from Below: An Ethnography of Maternal Health Encounters in the Peruvian Andes, The University of Liverpool (United Kingdom), 2018.

[daag082-B55] Morales GE . There is No place like home: imitation and the politics of recognition in Bolivian obstetric care. Med Anthropol Q 2018;32:404–24. 10.1111/maq.12427 29344977

[daag082-B56] Munn Z, Peters MD, Stern C et al Systematic review or scoping review? Guidance for authors when choosing between a systematic or scoping review approach. BMC Med Res Methodol 2018;18:143. 10.1186/s12874-018-0611-x 30453902 PMC6245623

[daag082-B57] Musie MR, Mulaudzi MF, Anokwuru R et al Recognise and acknowledge us: views of traditional birth attendants on collaboration with midwives for maternal health care services. Int J Reprod Med 2022;2022:9216500. 10.1155/2022/9216500 35874464 PMC9300345

[daag082-B58] Nacht A, Rivera C, Montes SB et al The addition of traditional birth attendant care to a home-based skilled nursing program in rural Guatemala: a secondary analysis from a quality improvement database. J Midwifery Women’s Health 2022;67:107–13. 10.1111/jmwh.13307 35060659 PMC8816827

[daag082-B59] Nureña CR . Incorporación del enfoque intercultural en el sistema de salud peruano: la atención del parto vertical. Rev Panam Salud Publica 2009;26:368–76. 10.1590/S1020-49892009001000013 20107687

[daag082-B60] Olaza-Maguiña AF, De La Cruz-Ramirez YM. Factors associated with negative birth experience in Peruvian Quechua-speaking indigenous women in a context of contagion due to COVID-19. Int J Gynaecol Obstet 2024;164:633–40. 10.1002/ijgo.15219 37922212

[daag082-B61] Olivas ET, Valdez M, Muffoletto B et al Reducing inequities in maternal and child health in rural Guatemala through the CBIO+ Approach of Curamericas: 6. Management of pregnancy complications at Community Birthing Centers (Casas Maternas Rurales). Int J Equity Health 2023;21:204. 10.1186/s12939-022-01758-6 36855147 PMC9976365

[daag082-B62] Organización Panamericana de la Salud . Una visión de salud intercultural para los pueblos indígenas de las Américas. Washington, DC: OPS, 2008. 10.26633/RPSP.2022.82 PMC9299388 35875317

[daag082-B63] Ortiz Anaya Y, Guillermo Rojas J. Cultural care practices provided at home by the Zenú Indigenous mothers to their premature children and to those with low birth weight. Invest Educ Enferm 2022;40:e09. 10.17533/udea.iee.v40n2e09 PMC9714986 36264697

[daag082-B64] Ouzzani M, Hammady H, Fedorowicz Z et al Rayyan—a web and mobile app for systematic reviews. Syst Rev 2016;5:210. 10.1186/s13643-016-0384-4 27919275 PMC5139140

[daag082-B65] Pan American Health Organization . Policy on Ethnicity and Health. Governing Bodies Documents. Washington, DC: PAHO, 2017.

[daag082-B66] Pan American Health Organization . Tool for Promoting Culturally Safe Childbirth: Basic Manual. Washington, DC: PAHO, 2023.

[daag082-B67] Paulino NA, Vázquez MS, Bolúmar F. Indigenous language and inequitable maternal health care, Guatemala, Mexico, Peru and the Plurinational State of Bolivia. Bull World Health Organ 2019;97:59–67. 10.2471/BLT.18.216184 30618466 PMC6307509

[daag082-B68] Penn-Kekana L, Pereira S, Hussein J et al Understanding the implementation of maternity waiting homes in low- and middle-income countries: a qualitative thematic synthesis. BMC Pregnancy Childbirth 2017;17:269. 10.1186/s12884-017-1444-z 28854880 PMC5577673

[daag082-B69] Peters MDJ, Godfrey C, McInerney P et al Scoping reviews. JBI Manual Evid Synth 2020a;10:10-46658. 10.46658/JBIMES-24-09 33038124

[daag082-B70] Peters MDJ, Marnie C, Tricco AC et al Updated methodological guidance for the conduct of scoping reviews. JBI Evid Synth 2020b;18:2119–26. 10.11124/JBIES-20-00167 33038124

[daag082-B71] Pulido Hernández Y . Los espacios interculturales en salud materna: Una oportunidad para entablar el diálogo intercultural. Carta Tepa Mayo 4 2022;1:87–113. 10.32870/ctm4.v1i7.42

[daag082-B72] Radoff KA, Levi AJ, Thompson LM. A radio-education intervention to improve maternal knowledge of obstetric danger signs. Rev Panam Salud Publica 2013;34:213–9. https://www.scielosp.org/pdf/rpsp/2013.v34n4/213-219/en 24301731

[daag082-B73] Rangel Flores YY, Hernández Ibarra LE, González Acevedo CE et al Agenciamientos y resistencias en el cuidado obstétrico comunitario tras la capacitación institucional. Index de Enfermería 2017;26:250–4. https://scielo.isciii.es/scielo.php?script=sci_arttext&pid=S1132-12962017000300003

[daag082-B74] Rojas IC . Estudio del servicio de salud materna en el hospital general del distrito de Jaén, brindado a través del Seguro Integral de Salud: Análisis desde el enfoque intercultural durante los meses de marzo a setiembre de 2012, Pontificia Universidad Catolica del Peru (Peru), 2015.

[daag082-B75] Roosta-G M . Madres indígenas enfrentan más de tres demoras: Los desafíos de la interculturalidad en salud. J Selva Andina Res Soc 2015;6:64–74. 10.36610/j.jsars.2015.060200064

[daag082-B76] Ruiz MJ, van Dijk MG, Berdichevsky K et al Barriers to the use of maternity waiting homes in indigenous regions of Guatemala: a study of users’ and community members’ perceptions. Cult Health Sex 2013;15:205–18. 10.1080/13691058.2012.751128 23234509

[daag082-B77] Rutledge JD, Kiyanda A, Jean-Louis C et al Recommendations for integrating traditional birth attendants to improve maternal health outcomes in low-and middle-income countries. Int J MCH AIDS 2024;13:e019. 10.25259/IJMA_16_2024 39526165 PMC11544515

[daag082-B78] Sáenz R, Nigenda G, Gómez-Duarte I et al Persistent inequities in maternal mortality in Latin America and the Caribbean, 1990–2019. Int J Equity Health 2024;23:96. 10.1186/s12939-024-02100-y 38730305 PMC11088099

[daag082-B79] Samuel J . The role of civil society in strengthening intercultural maternal health care in local health facilities: Puno, Peru. Glob Health Action 2016;9:33355. 10.3402/gha.v9.33355 27987298 PMC5161796

[daag082-B80] Santana Mera LJ, Boza AJV. Estrategia de Integración al Sistema de Salud Pública Ecuatoriano de Parteras/Os, Parroquia Quisapincha, Ambato, Universidad Técnica de Ambato, Ecuador, 2016.

[daag082-B81] Sarmiento I, Paredes-Solís S, de Jesús García A et al Safe birth in cultural safety in southern Mexico: a pragmatic non-inferiority cluster-randomised controlled trial. BMC Pregnancy Childbirth 2022;22:43. 10.1186/s12884-021-04344-w 35038990 PMC8762841

[daag082-B82] Sarmiento I, Paredes-Solís S, De Jesús-García A et al Traditional midwifery contribution to safe birth in cultural safety: narrative evaluation of an intervention in Guerrero, Mexico. Community Health Equity Res Policy 2024;44:377–89. 10.1177/0272684X221120481 36189713 PMC11143758

[daag082-B83] Sarmiento I, Paredes-Solís S, Dion A et al Maternal health and Indigenous traditional midwives in southern Mexico: contextualisation of a scoping review. BMJ Open 2021;11:e054542. 10.1136/bmjopen-2021-054542 PMC8710897 34949629

[daag082-B84] Sarmiento I, Paredes-Solís S, Loutfi D et al Fuzzy cognitive mapping and soft models of Indigenous knowledge on maternal health in Guerrero, Mexico. BMC Med Res Methodol 2020a;20:125. 10.1186/s12874-020-00998-w 32429974 PMC7238543

[daag082-B85] Sarmiento I, Zuluaga G, Paredes-Solís S et al Bridging Western and Indigenous knowledge through intercultural dialogue: lessons from participatory research in Mexico. BMJ Glob Health 2020b;5:e002488. 10.1136/bmjgh-2020-002488 PMC7526303 32994227

[daag082-B86] Selbana DW, Derese M, Sewmehone Endalew E et al A culturally sensitive and supportive maternity care service increases the uptake of maternity waiting homes in Ethiopia. Int J Women’s Health 2020;12:813–21. 10.2147/IJWH.S268245 33116931 PMC7553138

[daag082-B87] Shaikh BT, Khan S, Maab A et al Emerging role of traditional birth attendants in mountainous terrain: a qualitative exploratory study from Chitral District, Pakistan. BMJ Open 2014;4:e006238. 10.1136/bmjopen-2014-006238 PMC4248099 25428631

[daag082-B88] Sibley LM, Sipe TA, Barry D. Traditional birth attendant training for improving health behaviours and pregnancy outcomes. Cochrane Database Syst Rev 2012;2012:CD005460. 10.1002/14651858.CD005460.pub3 PMC4158424 22895949

[daag082-B89] Solís MPA, Puschner SMC, Ibarra VV et al Experiencias locales de salud materna con perspectiva intercultural en Putre y Tirúa, Chile. Desacatos. Revista de Ciencias Sociales 2023;71:130–45.

[daag082-B90] Sousa MF, Corning-Davis B. Building capacity to provide maternal health care in an Indigenous Guatemalan community through ultrasound and skills training. J Radiol Nurs 2019;38:123–30. 10.1016/j.jradnu.2019.03.002

[daag082-B91] Souza JP, Day LT, Rezende-Gomes AC et al A global analysis of the determinants of maternal health and transitions in maternal mortality. Lancet Glob Health 2024;12:e306–16. 10.1016/S2214-109X(23)00468-0 38070536

[daag082-B92] Stokes G, Sutcliffe K, Thomas J. Is a one-size-fits-all ‘12-month rule’appropriate when it comes to the last search date in systematic reviews? BMJ Evid Based Med 2023;28:359–63. 10.1136/bmjebm-2022-112060 PMC10715459 36600443

[daag082-B93] Stollak I, Valdez M, Rivas K et al Casas maternas in the rural highlands of Guatemala: a mixed-methods case study of the Introduction and utilization of birthing facilities by an Indigenous population. Glob Health Sci Pract 2016;4:114–31. 10.9745/GHSP-D-15-00266 27016548 PMC4807753

[daag082-B94] Stroux L, Martinez B, Coyote Ixen E et al An mHealth monitoring system for traditional birth attendant-led antenatal risk assessment in rural Guatemala. J Med Eng Technol 2016;40:356–71. 10.1080/03091902.2016.1223196 27696915 PMC5180361

[daag082-B95] Suárez-Baquero DFM, Champion JD. Traditional partería providing women’s health care in Latin America: a qualitative synthesis. Int Nurs Rev 2021;68:533–42. 10.1111/inr.12719 34624933

[daag082-B96] Summer A, Guendelman S, Kestler E et al Professional midwifery in Guatemala: a qualitative exploration of perceptions, attitudes and expectations among stakeholders. Soc Sci Med 2017;184:99–107. 10.1016/j.socscimed.2017.05.005 28511055

[daag082-B97] Summer A, Walker D. Recommendations for sustainable midwifery in Guatemala. World Med Health Policy 2018;10:356–80. 10.1002/wmh3.282

[daag082-B98] Summer A, Walker D, Guendelman S. A review of the forces influencing maternal health policies in post-war Guatemala. World Med Health Policy 2019;11:59–82. 10.1002/wmh3.292

[daag082-B99] Torri MC . Perceptions and uses of plants for reproductive health among traditional midwives in Ecuador: moving towards intercultural pharmacological practices. Midwifery 2013;29:809–17. 10.1016/j.midw.2012.06.018 22877763

[daag082-B100] Torri MC, Hollenberg D. Indigenous traditional medicine and intercultural healthcare in Bolivia: a case study from the Potosi region. J Community Health Nurs 2013;30:216–29. 10.1080/07370016.2013.838495 24219641

[daag082-B101] Tovilla GA . Impacto de la aplicación de la sala de atención al parto intercultural SNAIL BU CHVOK OLOL en las tasas de mortalidad materna del Centro de Salud CHIMIX DOS. Rev Latinoam Investig Educ 2022;1:18–25. https://unimeso.edu.mx/ojs/index.php/ReLIE/article/view/46

[daag082-B102] Trejos Serrato J . Experiência da equipe de enfermagem no cuidado às mulheres indígenas no processo de parto e nascimento em Cauca, Colômbia. Master's thesis, Universidade Federal do Paraná, Curitiba, Brazil, 2022.

[daag082-B103] Tricco AC, Lillie E, Zarin W et al PRISMA extension for scoping reviews (PRISMA-ScR): checklist and explanation. Ann Intern Med 2018;169:467–73. 10.7326/M18-0850 30178033

[daag082-B104] Tubino F . Entre el multiculturalismo y la interculturalidad: más allá de la discriminación positiva. Derecho & Sociedad 2002;19:299–311. https://revistas.pucp.edu.pe/index.php/derechoysociedad/article/view/17276

[daag082-B105] Tubino F . Del interculturalismo funcional al interculturalismo crítico. Rostros y fronteras de la identidad 2004;158:1–9. https://red.pucp.edu.pe/ridei/fixes/2011/08/1110.pdf

[daag082-B106] Tubino F . La Interculturalidad en Cuestión. Lima, Peru: Fondo Editorial de la PUCP, 2016.

[daag082-B107] Tucker K, Ochoa H, Garcia R et al The acceptability and feasibility of an intercultural birth center in the highlands of Chiapas, Mexico. BMC Pregnancy Childbirth 2013;13:94. 10.1186/1471-2393-13-94 23587122 PMC3679776

[daag082-B108] UNESCO . The 2005 Convention on the Protection and Promotion of the Diversity of Cultural Expressions. The General Conference of the United Nations Educational, Scientific and Cultural Organization. Paris. 33rd session, 2005.

[daag082-B109] UNICEF Argentina . Una iniciativa para proteger a las madres y sus bebés. 2023. https://www.unicef.org/argentina/historias/salud-materna-intercultural

[daag082-B110] van Braam EJ, McRae DN, Portela AG et al Stakeholders’ perspectives on the acceptability and feasibility of maternity waiting homes: a qualitative synthesis. Reprod Health 2023;20:101. 10.1186/s12978-023-01615-x 37407983 PMC10324180

[daag082-B111] van Dijk M, Ruiz MJ, Letona D et al Ensuring intercultural maternal health care for Mayan women in Guatemala: a qualitative assessment. Cult Health Sex 2013;15:S365–82. 10.1080/13691058.2013.779026 23713447

[daag082-B112] Vicente Martín P . Los estudios de salud materna intercultural en Bolivia de la teoría a la praxis. Boletín americanista 2017;74:91–111. https://revistes.ub.edu/index.php/BoletinAmericanista/article/view/17554

[daag082-B113] Vieira C, Portela A, Miller T et al Increasing the use of skilled health personnel where traditional birth attendants were providers of childbirth care: a systematic review. PLoS One 2012;7:e47946. 10.1371/journal.pone.0047946 23110138 PMC3480459

[daag082-B114] Vivar SC . Ecuador addresses cultural issues for pregnant women. The Lancet 2007;370:1302. 10.1016/S0140-6736(07)61561-X 17939187

[daag082-B115] Walsh SM, Norr KF, Sipsma H et al Effectiveness of a campaign to implement chlorhexidine use for newborns in rural Haiti. BMC Res Notes 2017;10:742. 10.1186/s13104-017-3059-x 29258564 PMC5735514

[daag082-B116] Walton A, Kestler E, Dettinger JC et al Impact of a low-technology simulation-based obstetric and newborn care training scheme on non-emergency delivery practices in Guatemala. Int J Gynaecol Obstet 2016;132:359–64. 10.1016/j.ijgo.2015.08.009 26797198 PMC4780429

[daag082-B117] Watson H, Harrop D, Walton E et al A systematic review of ethnic minority women’s experiences of perinatal mental health conditions and services in Europe. PLoS One 2019;14:e0210587. 10.1371/journal.pone.0210587 30695019 PMC6351025

[daag082-B118] Wight D, Wimbush E, Jepson R et al Six steps in quality intervention development (6SQuID). J Epidemiol Community Health 2016;70:520–5. 10.1136/jech-2015-205952 26573236 PMC4853546

[daag082-B119] Wikberg AM . A theory on intercultural caring in maternity care. Scand J Caring Sci 2021;35:442–56. 10.1111/scs.12856 32291776

[daag082-B120] Wilson A, Gallos ID, Plana N et al Effectiveness of strategies incorporating training and support of traditional birth attendants on perinatal and maternal mortality: meta-analysis. BMJ 2011;343:d7102. 10.1136/bmj.d7102 22134967 PMC3228291

[daag082-B121] World Bank Group . A Behavioral Approach to Uncover Barriers to Maternal Care in Haiti. eMBed brief Washington, DC: World Bank Group. 2021. http://documents.worldbank.org/curated/en/591881630339332207

[daag082-B122] World Health Organization . Ottawa Charter for Health Promotion, Vol. 21, 1986, 17–21. First International Conference on Health Promotion Ottawa, 21 November 1986 - WHO/HPR/HEP/95.1. 1986.

[daag082-B123] World Health Organization . Traditional Birth Attendants: a Joint WHO/UNFPA/UNICEF Statement. Geneva, Switzerland: World Health Organization, 1992.

[daag082-B124] World Health Organization . Everybody’s Business: Strengthening Health Systems to Improve Health Outcomes: WHO’s Framework for Action. Geneva, Switzerland: World Health Organization, 2007.

[daag082-B125] World Health Organization . Companion of Choice During Labour and Childbirth for Improved Quality of Care. Geneva, Switzerland: World Health Organization, 2016.

[daag082-B126] World Health Organization . Definition of Skilled Health Personnel Providing Care During Childbirth: the 2018 Joint Statement by WHO. Geneva, Switzerland: World Health Organization, 2018.

[daag082-B127] World Health Organization . Migration and Health: Enhancing Intercultural Competence and Diversity Sensitivity. Geneva, Switzerland: World Health Organization, 2020.

[daag082-B128] World Health Organization . Trends in Maternal Mortality 2000 to 2020: Estimates by WHO, UNICEF, UNFPA, World Bank Group and UNDESA/Population Division. Geneva, Switzerland: World Health Organization, 2023.

