# Supplementary material for: Navigating strategies for intercultural maternal and newborn care in Latin America and the Caribbean: a scoping review
Source: Health Promot Int. 2026 Jun 15;41(3):daag082. doi: 10.1093/heapro/daag082 (PMC13267143; doi:10.1093/heapro/daag082)
Supplement: daag082_Supplementary_Data [file daag082_supplementary_data.zip › Supplementary File 2_ Protocol.docx]

**Supplementary File 2: Protocol**

**Form used:** Van den Akker, O. R., Peters, G. Y., Bakker, C., Carlsson, R., Coles, N. A., Corker, K. S., Feldman, G., , Moreau, D., Nordström, T., Pfeiffer, N., Pickering, J. S., Riegelman, A., Topor, M., Veggel, N., Yeung, S., Mellor, D., & Pfeiffer, N. Generalized Systematic Review Registration Form. MetaArXiv. <https://doi.org/g5fj>

**Reference:** Vilchez, F. I., de Almeida, M. M. G., Filho, T. B. d. N., Angelovská, O., & Kotherová, Z. (2024, March 15). Navigating Strategies for Intercultural Maternal Care in Latin America: A Scoping Review. <https://doi.org/10.17605/OSF.IO/QSXK7>

**Review Methods**

In this section, you register the general type, background, and goals of your review.

**Type of review ***

| This scoping review will follow the Joanna Briggs Institute Manual for Scoping Reviews (Peters, 2020). The review report will follow the recommendations of Preferred Reporting Items for Systematic Reviews and Meta-Analyses - extension for scoping reviews (PRISMA-ScR) (Tricco, 2018). |
| --- |

**Review stages***

| Protocol Elaboration, Protocol Preregistration, Search, Screening, Extraction, Synthesis. |
| --- |

**Current review stage ***

| Protocol Preregistration. |
| --- |

**Start date ***

| 15 January, 2024 |
| --- |

**End date ***

| 15 June, 2024 |
| --- |

**Background ***

| In the last decade, global attention has been paid to maternal-neonatal health due to the high rates of mortality during pregnancy or postpartum (Fouard, 2019). In Latin America and the Caribbean, the maternal mortality ratio presents a regional average of 87 per 100,000 live births for 2020. This high ratio shows a public health problem in terms of quality, human rights, and development (MCLCP, 2021). According to the World Health Organization (WHO) (2023), in Latin America, approximately 30 women die from maternal-related events daily, events that are preventable with better access to care and good quality maternal-neonatal interventions. In this context, the challenge of maternal mortality is exacerbated by geographical heterogeneity; this allows us to visualize the situations of inequality in the response capacity of primary healthcare in the region (MCLCP, 2021; Guevara, 2016).  Globally, governments have undertaken diverse initiatives to ensure high-quality maternal and neonatal health services provision for mothers and children (Burgos, 2015; UNFPA, 2014). In Latin America, this provision faces more difficulties because of the coexistence between various indigenous communities that have their traditional health codes. Those codes are translated into ancestral practices like vertical births with the participation of a midwife, prenatal check-ups in the mother's tongue, delivery of the maternal placenta, maternity houses, and others (Richardson et al., 2020; Agramonte, 2016; Birth, 2018; Garbelli & Lira, 2021; Healy et al., 2020). In that way, traditional culturally sensitive practices are defined as intercultural maternal health services. The intercultural approach promotes dialogue and tolerance while maintaining cultural identity (Tubino, 2015), considering the beliefs, practices, and values of the women served to provide them with quality and culturally sensitive medical care.  In recent years, intercultural health initiatives have been verified at a global level with a synergy between cultural/traditional and hegemonic health. In other words, prenatal care has been applied in the indigenous mother tongue, respecting customs regarding pregnancy, access to maternity homes, vertical births in public health institutions with the participation of midwives, and delivery of the maternal placenta, all with an understanding of ancestral knowledge and community participation.  The potential for empowerment through the utilization of intercultural maternal health services aligns with the autonomy and agency of women who choose to access these services. In this sense, interculturality in health is a socially effective and humanely fair inter-learning that deserves deep attention in developing countries.  Strategies have been implemented in different Latin American countries to address cultural competence in maternal and newborn care. However, the literature from the last ten years has not been synthesized. Therefore, we aim to identify, categorize, and describe strategies for promoting intercultural maternal and newborn care in Latin America through a scoping review. |
| --- |

**Primary research question(s) ***

| The research question was structured using the acronym PCC as follows:  · P (population, condition): women during pregnancy, childbirth, and after birth or neonates.  · C (concept): strategies or interventions for promoting intercultural maternal and newborn care or culturally appropriate maternity care.  · C (context): Latin American countries.  *Intercultural care: Interculturality in health care is defined as the synergy or inter-learning between the traditional way of doing health and the institutional one. This synergy is considered socially effective and humanly fair for all populations, including the rural ones (Tubino, 2015).  *Latin America: Region in the Americas that includes Mexico, South America, Central America, and the Caribbean.  - South America: Argentina, Bolivia (Plurinational State of), Brazil, Chile, Colombia, Ecuador, French Guiana, Guyana, Paraguay, Peru, Suriname, Uruguay, Venezuela (the Bolivarian Republic of);  - Central America: Belize, Costa Rica, El Salvador, Guatemala, Honduras, Nicaragua, Panama;  - Caribbean: Anguilla, Antigua and Barbuda, Aruba, the Bahamas, Barbados, Bermuda, British Virgin Islands, Cayman Islands, Cuba, Dominica, Dominican Republic, Grenada, Jamaica, Haiti, Montserrat, Netherlands Antilles, Puerto Rico, St. Kitts and Nevis, Saint Lucia, St. Vincent and the Grenadines, Suriname, Trinidad and Tobago, Turks and Caicos and the United States Virgin Islands. |
| --- |

**Secondary research question(s) ***

| Not applicable. |
| --- |

**Expectations / hypotheses ***

| Not applicable. |
| --- |

**Dependent variable(s) / outcome(s) / main variables ***

| Not applicable. |
| --- |

**Independent variable(s) / intervention(s) / treatment(s) ***

| Not applicable. |
| --- |

**Additional variable(s) / covariate(s) ***

| Not applicable. |
| --- |

**Software ***

| · Storage and deduplication: Zotero version 6.0.30.  · Screening: Rayyan platform (Ouzzani 2016).  · Extracting data: Microsoft Excel® and STATA®.  · Synthesize the results: Microsoft Excel®, STATA® and Microsoft Word®. |
| --- |

**Funding ***

| At the time of this preregistration, this project is unfunded. |
| --- |

**Conflicts of interest ***

| This project has no conflict of interest. |
| --- |

**Overlapping authorships ***

| We do not expect any overlapping authorship. |
| --- |

**Search Strategy**

**Databases ***

| We will conduct a comprehensive, sensitive, and unrestricted search in the literature through structured search strategies, with relevant descriptors and synonyms, for the following databases:  · LILACS  · SCIELO  · Redalyc  · PubMed  · Web of Science  · ProQuest  · APA PsycInfo  · EMBASE  · Cochrane Library |
| --- |

**Interfaces ***

| · LILACS (via BVSalud)  · SCIELO  · Redalyc  · Medical Literature Analysis and Retrieval System Online (MEDLINE, via PubMed)  · Web of Science  · ProQuest  · APA PsycInfo (via EBSCOhost)  · EMBASE (via Elsevier)  · Cochrane Library (via Wiley) |
| --- |

**Grey literature ***

| We will perform searches for grey literature to identify theses and dissertations (ProQuest, World Health Organization, Pan American Health Organization). |
| --- |

**Inclusion and exclusion criteria ***

| Criteria for inclusion of studies according to the components of the PCC acronym  · P (population, condition): we will include studies that address women during pregnancy, childbirth, and postpartum or neonates in Latin America.  · C (concept): interventions or strategies for promoting intercultural maternal and newborn care or culturally appropriate maternity care. We will consider any strategy or program explicitly focusing on accommodating cultural groups' values, beliefs, practices, behaviors, norms, or language. Cultural groups include indigenous groups, tribal communities, and any form of group distinguished by its unique cultural traits. For that aim, we will consider as synonyms the following terms: intercultural, culturally appropriate, culturally competent, culturally sensitive, and culturally responsive care.  · C (context): we will include any strategy related to intercultural maternal and newborn care in the context of a healthcare unit, neighborhood, municipality, state, region, or country in Latin America.  We will consider any primary (descriptive or analytical) or secondary study design addressing interventions or strategies for promoting intercultural maternal and newborn care in Latin America in the context of public health.  Potentially eligible studies will include full publications, theses, and dissertations published in English, Spanish, or Portuguese.  We will consider studies published from 2013 to the present. The last broad literature mapping on this topic included items published from 1990 to 28 February 2013 (Coast, 2014).  We will include the following studies: research articles, reviews, conceptual articles, grey literature, trials, quasi-experimental, comparative, observational, and modeling.  We will exclude the following studies: policy documents, books, book chapters, case reports, editorials, commentaries, and conference abstracts. |
| --- |

**Query strings ***

| During this preregistration, we did not start the search strategy validation procedures. As a result, the current set of keywords is preliminary. In the subsequent iterative validation process, we will refine the search strategy to guarantee comprehensiveness.  · English  Interculturality terms  intercultural; interculturality. “Culturally-appropriate”; “culturally acceptable”; “culturally competent”; “cultural competence”; “culturally sensitive”; “cultural sensitivity”; “culturally responsive”; “culturally adapted”; “culturally safe”;“cultural safety”; “cultural care”; “cultural brokering”; “cultural broker”; “Traditional Birth Attendant”; “Traditional Birth Attendants”; “vertical birth”  Healthcare terms  Health; healthcare; care  Maternal/neonatal terms  Maternal; neonatal; perinatal; child; infant; infants; neonate; neonates; newborn; newborns; women; pregnancy; partum; birth; childbirth; antepartum; prenatal; prepartum; postpartum; postpartum; breastfeeding; midwifery; midwifes; midwives; reproductive  · Spanish  Interculturality terms  “apropiado culturalmente”; “aceptado culturalmente”; “culturalmente competente”; “culturalmente sensible”; “sensibilidad cultural”; “adaptación cultural”; “culturalmente seguro”; “cuidado cultural”; “intercultural”; “interculturalidad”; “partos tradicionales”; “salud cultural”; “ salud alternativa”; “partos verticales”  Healthcare terms  Salud; cuidado; atención  Maternal/neonatal terms  Materna; materno; neonata; materno-neonatal; perinatal; niño; infante; infantes; neonato; neonatos; “recién nacido”; “recién nacidos”; mujer; embarazada; embarazo; parto; nacimiento; prenatal; posparto; lactancia; partera; reproducción  · Portuguese  Interculturality terms  “Culturalmente apropriado”; “Culturalmente aceito”; “Culturalmente sensível”; “Culturalmente competente”; “Competência cultural”; “Adaptação cultural”; “Cuidado cultural”; Intercultural; Interculturalidade; Interétnica; Interétnicos; “Medicina tradicional”; “Parto vertical”  Healthcare terms  Assistência; cuidado; saúde; atenção  Maternal/neonatal terms  Materno; materna; gestante; gestação; mulher; anteparto; “pré-natal”; perinatal; parto; “pós-parto”; criança; crianças; bebês; “recém-nascido”; “recém-nascidos”; neonatal; neonato; neonatos; obstétrica; obstetrícia; amamentação; aleitamento; parteira; parteiras; doula; doulas; reprodutivo; reprodutiva |
| --- |

**Search validation procedure ***

| We will ensure the comprehensiveness of our search strategy through a validation procedure. Initially, we will identify pertinent, well-established studies for our scoping review. We will run the search strategy in the selected databases to ascertain the retrieval of these known papers. We will evaluate the extent to which the search strategy successfully captures these studies, noting any discrepancies or omissions. If our strategy misses known studies, we will refine it by adjusting search terms or parameters. This iterative validation process will continue until the search strategy retrieves all known relevant studies. |
| --- |

**Other search strategies ***

| Not applicable. |
| --- |

**Procedures to contact authors ***

| None. |
| --- |

**Results of contacting authors ***

| Not applicable. |
| --- |

**Search expiration and repetition ***

| There is no plan to repeat the search at this time. |
| --- |

**Search strategy justification ***

| We have selected a comprehensive set of databases. Our query strings will encompass relevant keywords and synonyms associated with intercultural maternal-neonatal health in Latin America. We will validate the search to ensure we cast a wide net to identify relevant studies. |
| --- |

**Miscellaneous search strategy details ***

| Not applicable. |
| --- |

**Screening**

**Screening stages ***

| The study selection process will be carried out in three sequential phases. Initially, we will identify and exclude the duplicates. Secondly, the titles and abstracts of all references retrieved through the search strategies will undergo screening. These references will be classified as either "potentially eligible" or "eliminated. The third phase will consist of an examination of the full text of the “potentially eligible” studies to determine their eligibility or exclusion, with justifications for each exclusion in this phase provided. Independent reviewers will conduct the screening process. A third reviewer will resolve discrepancies. The selection process will be presented through a PRISMA flowchart. |
| --- |

**Screened fields / blinding ***

| No fields are blinded. |
| --- |

**Used exclusion criteria ***

| · Research published before January 1, 2013.  · Studies without representation of women during pregnancy, childbirth, and postpartum or neonates in their samples.  · Research that does not address strategies or programs designed to promote intercultural care.  · Studies incorporating intercultural maternal and newborn care in contexts other than Latin American countries. |
| --- |

**Screener instructions ***

| Screeners will be provided with the protocol. |
| --- |

**Screening reliability ***

| The two phases will be conducted independently by two groups of authors. |
| --- |

**Screening reconciliation procedure ***

| Any disagreements in decisions to include or exclude studies will be solved by a third author. |
| --- |

**Sampling and sample size ***

| Not applicable. |
| --- |

**Screening procedure justification ***

| We will employ a two-round screening process—initially assessing titles and abstracts, followed by full-text evaluation. The initial screening efficiently eliminates obviously irrelevant studies, optimizing time and resources. Full-text reading allows a comprehensive assessment of potentially relevant sources. We defined inclusion and exclusion criteria aligned with the research question. Discrepancies between independent reviewers will be resolved through consensus or, if needed, the involvement of a third reviewer. This process ensures collective decision-making and minimizes bias. |
| --- |

**Data management and sharing ***

| Excel files will be uploaded to OSF. |
| --- |

**Miscellaneous screening details ***

| Not applicable. |
| --- |

**Extraction**

**Entities to extract ***

| Two reviewers will independently extract data identified in the included studies, and differences will be solved by consulting a third author. The following data will be collected for each included study:  · Category: birthing practices, newborn care practices, language used during care provision, and participatory approaches. New categories can be further incorporated to cover all strategies identified in this review.  · Strategy recipients: women during pregnancy, childbirth, or postpartum, family members, maternity care providers, and community leaders, among others.  · Cultural groups addressed: indigenous communities, ethnic groups, rural communities, among others.  · Attendants involved: doctors, nurses, midwives, traditional birth attendants, among others.  · Environment for strategy implementation: hospital, ambulatory, among others.  · Strategy approach: individual, group, or community.  · Institutions involved: governmental, non-governmental, among others.  · Country/locality for which the strategy was developed.  · Strategy content: single or multiple interventions.  · Strategy duration: continuous or temporary.  · Delivery format: face-to-face, virtual, or hybrid.  · Strategy status: proposed, executed, and not evaluated, or implemented and evaluated.  · Expected costs for implementing the strategy.  · Outcomes: the impact expected or evaluated by proponents.  · Barriers and facilitators identified for strategy implementation.  We will also collect the following data for each study: author, year of publication, publication type, study design, and strategy description. |
| --- |

**Extraction stages ***

| Data from the included studies will be independently extracted by two authors. |
| --- |

**Extractor instructions ***

| Extractors will be provided with the protocol. |
| --- |

**Extractor masking ***

| Masking will not be used. |
| --- |

**Extraction reliability ***

| Data from the included studies will be independently extracted by two authors. |
| --- |

**Extraction reconciliation procedure ***

| Information discrepancies will be solved through consensus or by consulting a third author. |
| --- |

**Extraction procedure justification ***

| Extraction categories were distinctly named and defined to reduce the risk of bias in extraction. |
| --- |

**Data management and sharing ***

| Not applicable. |
| --- |

**Miscellaneous extraction details ***

| Not applicable. |
| --- |

**Synthesis and Quality Assessment**

**Planned data transformations ***

| The strategies related to intercultural maternal and newborn care will be classified using the categories previously described. This qualitative synthesis will be presented using a narrative approach and in graphics or tables. Depending on the availability of information, descriptive statistics will be performed using Microsoft Excel®, Stata® or R software. |
| --- |

**Missing data ***

| Not applicable. |
| --- |

**Data validation ***

| Not applicable. |
| --- |

**Quality assessment ***

| Since this is a scoping review aiming to map available evidence, checklists or tools to assess the methodological quality of studies will not be applied, as recommended by Joanna Briggs Institute for scoping reviews (Peters 2020). |
| --- |

**Synthesis plan ***

| The strategies related to intercultural maternal and newborn care will be classified using the categories previously described. This qualitative synthesis will be presented using a narrative approach and in graphics or tables. Depending on the availability of information, descriptive statistics will be performed using Microsoft Excel®, and Stata®. |
| --- |

**Criteria for conclusions / inference criteria ***

If you plan to draw your conclusions based on pre-specified criteria (e.g., a minimal effect size of interest, a significance level, or a saturation point), list these here.

| Not applicable. |
| --- |

**Synthesist blinding ***

| Synthesists will not be blinded. |
| --- |

**Synthesis reliability ***

| The synthesis will be carried out by two independent synthesizers, working to collaboratively examine and summarize the results obtained from the studies included in the analysis. |
| --- |

**Synthesis reconciliation procedure ***

| Team meetings. |
| --- |

**Publication bias analyses ***

| Not applicable. |
| --- |

**Sensitivity analyses / robustness checks ***

| Not applicable. |
| --- |

**Synthesis procedure justification ***.

| The synthesis procedure is structured to ensure reliability, data integrity, and review comprehensiveness. |
| --- |

**Synthesis data management and sharing ***

| Not applicable. |
| --- |

**Miscellaneous synthesis details ***

| References  Agramonte, M. (2016). Vertical childbirth: does it favor a correct neonatal adaptation? Public health Mex vol.58 no.3  Birth, K. (2018). Vertical childbirth positions: a systematic review.  Burgos, Al. (2015). Políticas públicas en América Latina para la reducción de la mortalidad materna, 2009-2014. United Nations, serie Población y Desarrollo  Coast E, Jones E, Portela A, Lattof SR. Maternity care services and culture: a systematic global mapping of interventions. PloS one. 2014 Sep 30;9(9):e108130.  Fouard, A. (2019). Health System Access to Maternal and Child Health Services in Sierra Leone.  Garbelli, L., & Lira, V. (2021). Maternal positions during labour: midwives' knowledge and educational needs in Northern Italy.  Guevara, E. (2016). Estado actual de la mortalidad materna en el Perú \| Revista Peruana de Investigación Materno Perinatal. https://investigacionmaternoperinatal.inmp.gob.pe/index.php/rpinmp/article/view/155  Healy, M., Nyman, V., et al. (2020). How do midwives facilitate women to give birth during physiological second stage of labour? A systematic review. Jul 28;15(7): e0226502. DOI: 10.1371/journal.pone.0226502. eCollection 2020.  MCLCP. (2021). Perú: mortalidad materna sigue en aumento en el contexto de covid-19. un llamado a la acción. Alerta N° 1-2021-SC/ GT Salud-MCLCP.  Ouzzani M, Hammady H, Fedorowicz Z, Elmagarmid A. Rayyan—a web and mobile app for systematic reviews. Systematic reviews. 2016 Dec;5:1-0.  Peters MDJ, Godfrey C, McInerney P, Munn Z, Tricco AC, Khalil, H. Chapter 11: Scoping Reviews (2020 version). In: Aromataris E, Munn Z (Editors). JBI Manual for Evidence Synthesis, JBI, 2020  Richardson, D., Andrea, S., et al. (2020). Local management for the implementation and operation of the maternity home. Latina Women: A systematic review.  Tricco AC, Lillie E, Zarin W, O'Brien KK, Colquhoun H, Levac D, et al. PRISMA extension for scoping reviews (PRISMA-ScR): checklist and explanation. Ann Intern Med. 2018,169(7):467-473  Tubino, F. (2015). La interculturalidad en cuestión. Fondo Editorial de la Pontificia Universidad Católica del Perú.  UNFPA (2014), The Global Programme to Enhance Reproductive Health Commodity Security Annual Report 2013, United Nations Population Fund. |
| --- |
