# Supplementary material for: Navigating strategies for intercultural maternal and newborn care in Latin America and the Caribbean: a scoping review
Source: Health Promot Int. 2026 Jun 15;41(3):daag082. doi: 10.1093/heapro/daag082 (PMC13267143; doi:10.1093/heapro/daag082)
Supplement: daag082_Supplementary_Data [file daag082_supplementary_data.zip › Supplementary File 3_Search Strategy.docx]

**Supplementary File 3: Search strategy**

**Search Strategy Structure**

|  | **English terms** | **Spanish terms*** | **Portuguese terms*** |
| --- | --- | --- | --- |
| **Intercultural terms** | "culturally appropriate"  "culturally acceptable"  "culturally competent"  "cultural competence"  "culturally sensitive"  "cultural sensitivity"  "culturally responsive"  "culturally adapted"  "culturally safe"  "cultural safety"  "cultural care"  intercultural  interculturality  "cultural brokering’  "cultural broker"  "Traditional Birth Attendant"  "Traditional Birth Attendants"  "vertical birth"  "cultural appropriateness"  "cultural responsiveness"  "cultural adaptation"  "cultural imperatives"  "cultural belief"  "cultural beliefs”  "cultural practice"  "cultural practices” | “apropiado culturalmente”  “aceptado culturalmente”  “culturalmente competente”  “culturalmente sensible”  “sensibilidad cultural”  “adaptación cultural”  “culturalmente seguro”  “responsabilidad cultural”  “cuidado cultural”  “interculturalidad”  “parto tradicional”  “partos tradicionales”  “salud cultural”  “salud alternativa”  “parto vertical”  “partos verticales”  “creencias culturales”  “practicas culturales” | “Culturalmente apropriado”  “Culturalmente aceito”  “Culturalmente sensível”  “Culturalmente competente”  “Competência cultural”  “sensibilidade cultural”  “Adaptação cultural”  “culturalmente adaptado”  “responsabilidade cultural”  “culturalmente responsável”  “Cuidado cultural”  “saúde cultural”  Interculturalidade  “Medicina tradicional”  “Parto vertical”  “práticas culturais”  “crenças culturais” |
| **Healthcare terms** | health  healthcare  care | salud  cuidado  atención | assistência  saúde  atenção |
| **Maternal-neonatal terms** | maternal  neonatal  perinatal  child  children  childhood  pediatric  paediatric  infant  infants  neonate  neonates  newborn  newborns  baby  babies  mother  women  pregnancy  pregnancies  childbearing  partum  labor  labour  birth  birthing  cesarean  childbirth  delivery  antepartum  prenatal  prepartum  peripartum  postpartum  pospartum  breastfeeding  "breast feeding"  breastfed  "breast milk"  lactation  lactating  midwifery  midwifes  midwives  midwife  doula  doulas  reproductive  obstetric  obstetrics | materna  materno  neonata  “materno-neonatal”  perinatal  niño  niños  infante  infantes  neonato  neonatos  “recién nacido”  “recién nacidos”  mujer  mujeres  embarazada  embarazo  parto  nacimiento  prenatal  posparto  lactancia  partera  reproducción | materno  materna  gestante  gestação  mulher  mulheres  anteparto  “pré-natal”  perinatal  periparto  parto  “pós-parto”  criança  crianças  bebês  “recém-nascido”  “recém-nascidos”  neonatal  neonato  neonatos  obstétrica  obstetrícia  amamentação  aleitamento  parteira  parteiras  doula  doulas  reprodutivo  reprodutiva  obstetrícia |

*Applied in Latin American Databases LILACS, Scielo, and Redalyc

**MEDLINE Search Strategy**

| **Database:** MEDLINE (via Pubmed) |
| --- |
| **Date of search:** 27 March 2024 |
| **Filters applied:** 2013 to 2024; Title and Abstract |
| **Number of records:** 7,071 |
| **Search query:** ((("culturally appropriate"[Title/Abstract] OR "culturally acceptable"[Title/Abstract] OR "culturally competent"[Title/Abstract] OR "cultural competence"[Title/Abstract] OR "culturally sensitive"[Title/Abstract] OR "cultural sensitivity"[Title/Abstract] OR "culturally responsive"[Title/Abstract] OR "culturally adapted" OR"culturally safe"[Title/Abstract] OR "cultural safety"[Title/Abstract] OR "cultural care"[Title/Abstract] OR intercultural[Title/Abstract] OR interculturality[Title/Abstract] OR "cultural brokering" OR"cultural broker"[Title/Abstract] OR "Traditional Birth Attendant"[Title/Abstract] OR "Traditional Birth Attendants"[Title/Abstract] OR "vertical birth"[Title/Abstract] OR "cultural appropriateness"[Title/Abstract] OR "cultural responsiveness"[Title/Abstract] OR "cultural adaptation"[Title/Abstract] OR "cultural imperatives"[Title/Abstract] OR "cultural belief"[Title/Abstract] OR "cultural beliefs"[Title/Abstract] OR "cultural practice"[Title/Abstract] OR "cultural practices"[Title/Abstract])) AND ((health[Title/Abstract] OR healthcare[Title/Abstract] OR care[Title/Abstract]))) AND ((maternal[Title/Abstract] OR neonatal[Title/Abstract] OR perinatal[Title/Abstract] OR child[Title/Abstract] OR children[Title/Abstract] OR childhood[Title/Abstract] OR pediatric[Title/Abstract] OR paediatric[Title/Abstract] OR infant[Title/Abstract] OR infants[Title/Abstract] OR neonate[Title/Abstract] OR neonates[Title/Abstract] OR newborn[Title/Abstract] OR newborns[Title/Abstract] OR baby[Title/Abstract] OR babies[Title/Abstract] OR mother[Title/Abstract] OR women[Title/Abstract] OR pregnancy[Title/Abstract] OR pregnancies[Title/Abstract] OR childbearing[Title/Abstract] OR partum[Title/Abstract] OR labor[Title/Abstract] OR labour[Title/Abstract] OR birth[Title/Abstract] OR birthing[Title/Abstract] OR cesarean[Title/Abstract] OR childbirth[Title/Abstract] OR delivery[Title/Abstract] OR antepartum[Title/Abstract] OR prenatal[Title/Abstract] OR prepartum[Title/Abstract] OR peripartum[Title/Abstract] OR postpartum[Title/Abstract] OR pospartum[Title/Abstract] OR breastfeeding[Title/Abstract] OR "breast feeding"[Title/Abstract] OR breastfed[Title/Abstract] OR "breast milk"[Title/Abstract] OR lactation[Title/Abstract] OR lactating[Title/Abstract] OR midwifery[Title/Abstract] OR midwifes[Title/Abstract] OR midwives[Title/Abstract] OR midwife[Title/Abstract] OR doula[Title/Abstract] OR doulas[Title/Abstract] OR reproductive[Title/Abstract] OR obstetric[Title/Abstract] OR obstetrics[Title/Abstract])) |

**Embase Search Strategy**

| **Database:** Embase |
| --- |
| **Date of search:** 27 March 2024 |
| **Filters applied:** 2013 to 2024; :ti,ab,kw |
| **Number of records:** 8,773 |
| **Search query:** (‘culturally appropriate’:ti,ab,kw OR ‘culturally acceptable’:ti,ab,kw OR ‘culturally competent’:ti,ab,kw OR ‘cultural competence’:ti,ab,kw OR ‘culturally sensitive’:ti,ab,kw OR ‘cultural sensitivity’:ti,ab,kw OR ‘culturally responsive’:ti,ab,kw OR ‘culturally adapted’:ti,ab,kw OR ‘culturally safe’:ti,ab,kw OR ‘cultural safety’:ti,ab,kw OR ‘cultural care’:ti,ab,kw OR ‘intercultural’:ti,ab,kw OR ‘interculturality’:ti,ab,kw OR ‘cultural brokering’:ti,ab,kw OR ‘cultural broker’:ti,ab,kw OR ‘Traditional Birth Attendant’:ti,ab,kw OR ‘Traditional Birth Attendants’:ti,ab,kw OR ‘vertical birth’:ti,ab,kw OR ‘cultural appropriateness’:ti,ab,kw OR ‘cultural responsiveness’:ti,ab,kw OR ‘cultural adaptation’:ti,ab,kw OR ‘cultural imperatives’:ti,ab,kw OR ‘cultural belief’:ti,ab,kw OR ‘cultural beliefs’:ti,ab,kw OR ‘cultural practice’:ti,ab,kw OR ‘cultural practices’:ti,ab,kw) **AND** (health:ti,ab,kw OR healthcare:ti,ab,kw OR care:ti,ab,kw) **AND** (maternal:ti,ab,kw OR neonatal:ti,ab,kw OR perinatal:ti,ab,kw OR child:ti,ab,kw OR children:ti,ab,kw OR childhood:ti,ab,kw OR pediatric:ti,ab,kw OR paediatric:ti,ab,kw OR infant:ti,ab,kw OR infants:ti,ab,kw OR neonate:ti,ab,kw OR neonates:ti,ab,kw OR newborn:ti,ab,kw OR newborns:ti,ab,kw OR baby:ti,ab,kw OR babies:ti,ab,kw OR mother:ti,ab,kw OR women:ti,ab,kw OR pregnancy:ti,ab,kw OR pregnancies:ti,ab,kw OR childbearing:ti,ab,kw OR partum:ti,ab,kw OR labor:ti,ab,kw OR labour:ti,ab,kw OR birth:ti,ab,kw OR birthing:ti,ab,kw OR cesarean:ti,ab,kw OR childbirth:ti,ab,kw OR delivery:ti,ab,kw OR antepartum:ti,ab,kw OR prenatal:ti,ab,kw OR prepartum:ti,ab,kw OR peripartum:ti,ab,kw OR postpartum:ti,ab,kw OR pospartum:ti,ab,kw OR breastfeeding:ti,ab,kw OR “breast feeding”:ti,ab,kw OR breastfed:ti,ab,kw OR “breast milk”:ti,ab,kw OR lactation:ti,ab,kw OR lactating:ti,ab,kw OR midwifery:ti,ab,kw OR midwifes:ti,ab,kw OR midwives:ti,ab,kw OR midwife:ti,ab,kw OR doula:ti,ab,kw OR doulas:ti,ab,kw OR reproductive:ti,ab,kw OR obstetric:ti,ab,kw OR obstetrics:ti,ab,kw) |

**Cochrane Library Search Strategy**

| **Database:** Cochrane Library |
| --- |
| **Date of search:** 27 March 2024 |
| **Filters applied:** Jan 2013 to Apr 2024; Title, abstract, keywords |
| **Number of records:** 4,961 |
| **Search query:** (culturally OR cultural OR culture OR intercultural OR interculturality OR “Traditional Birth Attendant” OR “Traditional Birth Attendants” OR “vertical birth”) **AND** (health OR healthcare OR care) **AND** (maternal OR neonatal OR perinatal OR child OR children OR childhood OR pediatric OR paediatric OR infant OR infants OR neonate OR neonates OR newborn OR newborns OR baby OR babies **OR** mother OR women OR pregnancy OR pregnancies OR childbearing OR partum OR labor OR labour OR birth OR birthing OR cesarean OR childbirth OR delivery OR antepartum OR prenatal OR prepartum OR peripartum OR postpartum OR pospartum OR breastfeeding OR “breast feeding” OR breastfed OR “breast milk” OR lactation OR lactating OR midwifery OR midwifes OR midwives OR midwife OR doula OR doulas OR reproductive OR obstetric OR obstetrics) |
| **Justification of search query adaptation:** the original search query did not retrieve any results. Therefore, we used broader terms to retrieve more results: culturally, cultural, and culture. |

**Web of Science Search Strategy**

| **Database:** Web of Science |
| --- |
| **Date of search:** 27 March 2024 |
| **Filters applied:** Date range 2013-2024; Topic |
| **Number of records:** 9,744 |
| **Search query:** (“culturally appropriate” OR “culturally acceptable” OR “culturally competent” OR “cultural competence” OR “culturally sensitive” OR “cultural sensitivity” OR “culturally responsive” OR “culturally adapted” OR“culturally safe” OR “cultural safety” OR “cultural care” OR intercultural OR interculturality OR “cultural brokering” OR“cultural broker” OR “Traditional Birth Attendant” OR “Traditional Birth Attendants” OR “vertical birth” OR “cultural appropriateness” OR “cultural responsiveness” OR “cultural adaptation” OR “cultural imperatives” OR “cultural belief” OR “cultural beliefs” OR “cultural practice” OR “cultural practices”) **AND** (health OR healthcare OR care) **AND** (maternal OR neonatal OR perinatal OR child OR children OR childhood OR pediatric OR paediatric OR infant OR infants OR neonate OR neonates OR newborn OR newborns OR baby OR babies **OR** mother OR women OR pregnancy OR pregnancies OR childbearing OR partum OR labor OR labour OR birth OR birthing OR cesarean OR childbirth OR delivery OR antepartum OR prenatal OR prepartum OR peripartum OR postpartum OR pospartum OR breastfeeding OR “breast feeding” OR breastfed OR “breast milk” OR lactation OR lactating OR midwifery OR midwifes OR midwives OR midwife OR doula OR doulas OR reproductive OR obstetric OR obstetrics) |

**ProQuest Search Strategy**

| **Database:**ProQuest |
| --- |
| **Date of search:** 27 March 2024 |
| **Filters applied:** Date range 2013-2024; Abstract |
| **Number of records:** 2.237 |
| **Search query:** abstract(("culturally appropriate" OR "culturally acceptable" OR "culturally competent" OR "cultural competence" OR "culturally sensitive" OR "cultural sensitivity" OR "culturally responsive" OR "culturally adapted" OR "culturally safe" OR "cultural safety" OR "cultural care" OR intercultural OR interculturality OR "cultural brokering" OR "cultural broker" OR "Traditional Birth Attendant" OR "Traditional Birth Attendants" OR "vertical birth" OR "cultural appropriateness" OR "cultural responsiveness" OR "cultural adaptation" OR "cultural imperatives" OR "cultural belief" OR "cultural beliefs" OR "cultural practice" OR "cultural practices") AND (health OR healthcare OR care) AND (maternal OR neonatal OR perinatal OR child OR children OR childhood OR pediatric OR paediatric OR infant OR infants OR neonate OR neonates OR newborn OR newborns OR baby OR babies OR mother OR women OR pregnancy OR pregnancies OR childbearing OR partum OR labor OR labour OR birth OR birthing OR cesarean OR childbirth OR delivery OR antepartum OR prenatal OR prepartum OR peripartum OR postpartum OR pospartum OR breastfeeding OR "breast feeding" OR breastfed OR "breast milk" OR lactation OR lactating OR midwifery OR midwifes OR midwives OR midwife OR doula OR doulas OR reproductive OR obstetric OR obstetrics)) OR title(("culturally appropriate" OR "culturally acceptable" OR "culturally competent" OR "cultural competence" OR "culturally sensitive" OR "cultural sensitivity" OR "culturally responsive" OR "culturally adapted" OR "culturally safe" OR "cultural safety" OR "cultural care" OR intercultural OR interculturality OR "cultural brokering" OR "cultural broker" OR "Traditional Birth Attendant" OR "Traditional Birth Attendants" OR "vertical birth" OR "cultural appropriateness" OR "cultural responsiveness" OR "cultural adaptation" OR "cultural imperatives" OR "cultural belief" OR "cultural beliefs" OR "cultural practice" OR "cultural practices") AND (health OR healthcare OR care) AND (maternal OR neonatal OR perinatal OR child OR children OR childhood OR pediatric OR paediatric OR infant OR infants OR neonate OR neonates OR newborn OR newborns OR baby OR babies OR mother OR women OR pregnancy OR pregnancies OR childbearing OR partum OR labor OR labour OR birth OR birthing OR cesarean OR childbirth OR delivery OR antepartum OR prenatal OR prepartum OR peripartum OR postpartum OR pospartum OR breastfeeding OR "breast feeding" OR breastfed OR "breast milk" OR lactation OR lactating OR midwifery OR midwifes OR midwives OR midwife OR doula OR doulas OR reproductive OR obstetric OR obstetrics)) |

**APA PsycInfo Search Strategy**

| **Database:** APA PsycInfo |
| --- |
| **Date of search:** 18 April 2024 |
| **Filters applied:** Date range 2013-2024 |
| **Number of records:** 20 |
| **Search query:** (“culturally appropriate” OR “culturally acceptable” OR “culturally competent” OR “cultural competence” OR “culturally sensitive” OR “cultural sensitivity” OR “culturally responsive” OR “culturally safe” OR “cultural safety” OR “cultural care” OR intercultural OR interculturality OR “vertical birth” OR “cultural appropriateness” OR “cultural responsiveness”) **AND** (health OR healthcare OR care) **AND** (maternal OR neonatal OR perinatal OR neonate OR neonates OR newborn OR newborns OR baby OR babies **OR** mother OR women OR pregnancy OR pregnancies OR childbearing OR partum OR labor OR labour OR birth OR birthing OR cesarean OR childbirth OR delivery OR antepartum OR antenatal OR prenatal OR prepartum OR peripartum OR postpartum OR pospartum OR breastfeeding OR “breast feeding” OR breastfed OR “breast milk” OR lactation OR lactating OR midwifery OR midwifes OR midwives OR midwife OR doula OR doulas OR reproductive OR obstetric OR obstetrics) |
| **Justification of search query adaptation:** we opted for a more restricted version since the original retrieved an unmanageable number of unrelated results. |

**LILACS Search Strategy**

| **Database:** LILACS |
| --- |
| **Date of search:** 27 March 2024 |
| **Filters applied:** 2013-2024; Título, resumo, assunto |
| **Number of records:** 1,172 |
| **Search query:** "culturally appropriate" OR "culturally acceptable" OR "culturally competent" OR "cultural competence" OR "culturally sensitive" OR "cultural sensitivity" OR "culturally responsive" OR "culturally adapted" OR "culturally safe" OR "cultural safety" OR "cultural care" OR intercultural OR interculturality OR "cultural brokering" OR "cultural broker" OR "Traditional Birth Attendant" OR "Traditional Birth Attendants" OR "vertical birth" OR "cultural appropriateness" OR "cultural responsiveness" OR "cultural adaptation" OR "cultural imperatives" OR "cultural belief" OR "cultural beliefs" OR "cultural practice" OR "cultural practices" **OR** “apropiado culturalmente” OR “aceptado culturalmente” OR “culturalmente competente” OR “culturalmente sensible” OR “sensibilidad cultural” OR “adaptación cultural” OR “culturalmente seguro” OR “responsabilidad cultural” OR “cuidado cultural” OR “interculturalidad” OR “parto tradicional” OR “partos tradicionales” OR “salud cultural” OR “salud alternativa” OR “parto vertical” OR “partos verticales” OR “creencias culturales” OR “practicas culturales” **OR** “Culturalmente apropriado” OR “Culturalmente aceito” OR “Culturalmente sensível” OR “Culturalmente competente” OR “Competência cultural” OR “sensibilidade cultural” OR “Adaptação cultural” OR “culturalmente adaptado” OR “responsabilidade cultural” OR “culturalmente responsável” OR “Cuidado cultural” OR “saúde cultural” OR Interculturalidade OR “Medicina tradicional” OR “Parto vertical” OR “práticas culturais” OR “crenças culturais” **AND** (health OR healthcare OR care OR salud OR cuidado OR atención OR assistência OR saúde OR atenção) AND (maternal OR neonatal OR perinatal OR child OR children OR childhood OR pediatric OR paediatric OR infant OR infants OR neonate OR neonates OR newborn OR newborns OR baby OR babies **OR** mother OR women OR pregnancy OR pregnancies OR childbearing OR partum OR labor OR labour OR birth OR birthing OR cesarean OR childbirth OR delivery OR antepartum OR prenatal OR prepartum OR peripartum OR postpartum OR pospartum OR breastfeeding OR “breast feeding” OR breastfed OR “breast milk” OR lactation OR lactating OR midwifery OR midwifes OR midwives OR midwife OR doula OR doulas OR reproductive OR obstetric OR obstetrics OR materna OR materno OR neonata OR “materno-neonatal” OR perinatal OR niño OR niños OR infante OR infantes OR neonato OR neonatos OR “recién nacido” OR “recién nacidos” OR mujer OR mujeres OR embarazada OR embarazo OR parto OR nacimiento OR prenatal OR posparto OR lactancia OR partera OR reproducción OR materno OR materna OR gestante OR gestação OR mulher OR mulheres OR anteparto OR “pré-natal” OR perinatal OR periparto OR parto OR “pós-parto” OR criança OR crianças OR bebês OR “recém-nascido” OR “recém-nascidos” OR neonatal OR neonato OR neonatos OR obstétrica OR obstetrícia OR amamentação OR aleitamento OR parteira OR parteiras OR doula OR doulas OR reprodutivo OR reprodutiva OR obstetrícia) |

**SciELO Search Strategy**

| **Database:** SciELO |
| --- |
| **Date of search:** 27 March 2024 |
| **Filters applied:** 2013-2024; Title OR Abstract |
| **Number of records:** 368 |
| **Search query:** (ti:(("culturally appropriate" OR "culturally acceptable" OR "culturally competent" OR "cultural competence" OR "culturally sensitive" OR "cultural sensitivity" OR "culturally responsive" OR "culturally adapted" OR "culturally safe" OR "cultural safety" OR "cultural care" OR intercultural OR interculturality OR "cultural brokering" OR "cultural broker" OR "Traditional Birth Attendant" OR "Traditional Birth Attendants" OR "vertical birth" OR "cultural appropriateness" OR "cultural responsiveness" OR "cultural adaptation" OR "cultural imperatives" OR "cultural belief" OR "cultural beliefs" OR "cultural practice" OR "cultural practices" OR “apropiado culturalmente” OR “aceptado culturalmente” OR “culturalmente competente” OR “culturalmente sensible” OR “sensibilidad cultural” OR “adaptación cultural” OR “culturalmente seguro” OR “responsabilidad cultural” OR “cuidado cultural” OR “interculturalidad” OR “parto tradicional” OR “partos tradicionales” OR “salud cultural” OR “salud alternativa” OR “parto vertical” OR “partos verticales” OR “creencias culturales” OR “practicas culturales” OR “Culturalmente apropriado” OR “Culturalmente aceito” OR “Culturalmente sensível” OR “Culturalmente competente” OR “Competência cultural” OR “sensibilidade cultural” OR “Adaptação cultural” OR “culturalmente adaptado” OR “responsabilidade cultural” OR “culturalmente responsável” OR “Cuidado cultural” OR “saúde cultural” OR Interculturalidade OR “Medicina tradicional” OR “Parto vertical” OR “práticas culturais” OR “crenças culturais”) AND (health OR healthcare OR care OR salud OR cuidado OR atención OR assistência OR saúde OR atenção) AND (maternal OR neonatal OR perinatal OR child OR children OR childhood OR pediatric OR paediatric OR infant OR infants OR neonate OR neonates OR newborn OR newborns OR baby OR babies OR mother OR women OR pregnancy OR pregnancies OR childbearing OR partum OR labor OR labour OR birth OR birthing OR cesarean OR childbirth OR delivery OR antepartum OR prenatal OR prepartum OR peripartum OR postpartum OR pospartum OR breastfeeding OR “breast feeding” OR breastfed OR “breast milk” OR lactation OR lactating OR midwifery OR midwifes OR midwives OR midwife OR doula OR doulas OR reproductive OR obstetric OR obstetrics OR materna OR materno OR neonata OR “materno-neonatal” OR perinatal OR niño OR niños OR infante OR infantes OR neonato OR neonatos OR “recién nacido” OR “recién nacidos” OR mujer OR mujeres OR embarazada OR embarazo OR parto OR nacimiento OR prenatal OR posparto OR lactancia OR partera OR reproducción OR materno OR materna OR gestante OR gestação OR mulher OR mulheres OR anteparto OR “pré-natal” OR perinatal OR periparto OR parto OR “pós-parto” OR criança OR crianças OR bebês OR “recém-nascido” OR “recém-nascidos” OR neonatal OR neonato OR neonatos OR obstétrica OR obstetrícia OR amamentação OR aleitamento OR parteira OR parteiras OR doula OR doulas OR reprodutivo OR reprodutiva OR obstetrícia))) OR (ab:(("culturally appropriate" OR "culturally acceptable" OR "culturally competent" OR "cultural competence" OR "culturally sensitive" OR "cultural sensitivity" OR "culturally responsive" OR "culturally adapted" OR "culturally safe" OR "cultural safety" OR "cultural care" OR intercultural OR interculturality OR "cultural brokering" OR "cultural broker" OR "Traditional Birth Attendant" OR "Traditional Birth Attendants" OR "vertical birth" OR "cultural appropriateness" OR "cultural responsiveness" OR "cultural adaptation" OR "cultural imperatives" OR "cultural belief" OR "cultural beliefs" OR "cultural practice" OR "cultural practices" OR “apropiado culturalmente” OR “aceptado culturalmente” OR “culturalmente competente” OR “culturalmente sensible” OR “sensibilidad cultural” OR “adaptación cultural” OR “culturalmente seguro” OR “responsabilidad cultural” OR “cuidado cultural” OR “interculturalidad” OR “parto tradicional” OR “partos tradicionales” OR “salud cultural” OR “salud alternativa” OR “parto vertical” OR “partos verticales” OR “creencias culturales” OR “practicas culturales” OR “Culturalmente apropriado” OR “Culturalmente aceito” OR “Culturalmente sensível” OR “Culturalmente competente” OR “Competência cultural” OR “sensibilidade cultural” OR “Adaptação cultural” OR “culturalmente adaptado” OR “responsabilidade cultural” OR “culturalmente responsável” OR “Cuidado cultural” OR “saúde cultural” OR Interculturalidade OR “Medicina tradicional” OR “Parto vertical” OR “práticas culturais” OR “crenças culturais”) AND (health OR healthcare OR care OR salud OR cuidado OR atención OR assistência OR saúde OR atenção) AND (maternal OR neonatal OR perinatal OR child OR children OR childhood OR pediatric OR paediatric OR infant OR infants OR neonate OR neonates OR newborn OR newborns OR baby OR babies OR mother OR women OR pregnancy OR pregnancies OR childbearing OR partum OR labor OR labour OR birth OR birthing OR cesarean OR childbirth OR delivery OR antepartum OR prenatal OR prepartum OR peripartum OR postpartum OR pospartum OR breastfeeding OR “breast feeding” OR breastfed OR “breast milk” OR lactation OR lactating OR midwifery OR midwifes OR midwives OR midwife OR doula OR doulas OR reproductive OR obstetric OR obstetrics OR materna OR materno OR neonata OR “materno-neonatal” OR perinatal OR niño OR niños OR infante OR infantes OR neonato OR neonatos OR “recién nacido” OR “recién nacidos” OR mujer OR mujeres OR embarazada OR embarazo OR parto OR nacimiento OR prenatal OR posparto OR lactancia OR partera OR reproducción OR materno OR materna OR gestante OR gestação OR mulher OR mulheres OR anteparto OR “pré-natal” OR perinatal OR periparto OR parto OR “pós-parto” OR criança OR crianças OR bebês OR “recém-nascido” OR “recém-nascidos” OR neonatal OR neonato OR neonatos OR obstétrica OR obstetrícia OR amamentação OR aleitamento OR parteira OR parteiras OR doula OR doulas OR reprodutivo OR reprodutiva OR obstetrícia))) AND (year_cluster:(2013 OR 2014 OR 2015 OR 2016 OR 2017 OR 2018 OR 2019 OR 2020 OR 2021 OR 2022 OR 2023 OR 2024)) |

**Redalyc Search Strategy**

| **Database:** Redalyc |
| --- |
| **Date of search:** 8 June 2024 |
| **Filters applied:** 2013-2024 |
| **Number of records:** 2555 |
| **Search query:** intercultural AND (maternal OR materna OR materno OR parto) AND (health) |
| **Justification of search query adaptation:** The initial comprehensive search strategy yielded over 450,000 articles. By scanning the search results, the research team identified that the majority were not pertinent to the research topic. To refine the search and ensure the triage process was manageable, we decided to include only terms directly related to our primary subject. When the keyword “intercultural” was used, Redalyc automatically included articles with related terms such as “interculturalidad.” After evaluating multiple search strategies, the research team selected the following search query: (intercultural AND (maternal OR materna OR materno OR parto) AND (health)). This search query was determined to provide an optimal balance between comprehensiveness and feasibility.  **Test 1: too broad, unfeasible**  (“apropiado culturalmente” OR “aceptado culturalmente” OR “culturalmente competente” OR “culturalmente sensible” OR “sensibilidad cultural” OR “adaptación cultural” OR “culturalmente seguro” OR “responsabilidad cultural” OR “cuidado cultural” OR “interculturalidad” OR “parto tradicional” OR “partos tradicionales” OR “salud cultural” OR “salud alternativa” OR “parto vertical” OR “partos verticales” OR “creencias culturales” OR “practicas culturales” OR “Culturalmente apropriado” OR “Culturalmente aceito” OR “Culturalmente sensível” OR “Culturalmente competente” OR “Competência cultural” OR “sensibilidade cultural” OR “Adaptação cultural” OR “culturalmente adaptado” OR “responsabilidade cultural” OR “culturalmente responsável” OR “Cuidado cultural” OR “saúde cultural” OR Interculturalidade OR “Medicina tradicional” OR “Parto vertical” OR “práticas culturais” OR “crenças culturais”) AND (salud OR cuidado OR atención OR assistência OR saúde OR atenção) AND (materna OR materno OR neonata OR “materno-neonatal” OR perinatal OR niño OR niños OR infante OR infantes OR neonato OR neonatos OR “recién nacido” OR “recién nacidos” OR mujer OR mujeres OR embarazada OR embarazo OR parto OR nacimiento OR prenatal OR posparto OR lactancia OR partera OR reproducción OR materno OR materna OR gestante OR gestação OR mulher OR mulheres OR anteparto OR “pré-natal” OR perinatal OR periparto OR parto OR “pós-parto” OR criança OR crianças OR bebês OR “recém-nascido” OR “recém-nascidos” OR neonatal OR neonato OR neonatos OR obstétrica OR obstetrícia OR amamentação OR aleitamento OR parteira OR parteiras OR doula OR doulas OR reprodutivo OR reprodutiva OR obstetrícia)  **Test 2: too restricted**  intercultural AND maternal AND health  288 results  **Test 3: too restricted**  intercultural AND maternal  570 results |

**Grey Literature and Snowballing**

| **Websites:**  WHO: https://www.who.int/publications/m  PAHO: https://iris.paho.org/  UNICEF: https://www.unicef.org/lac/publicaciones  World Bank: https://www.worldbank.org/ext/en/home  Google Scholar: https://scholar.google.com.br/  **Snowballing approach:** screening the references of included reviews |
| --- |
| **Period of search:** 14-18 October 2024 |
| **Filters applied:** 2013-2024; Title |
| **Number of records:**  World Bank, WHO, PAHO and UNICEF: 11  Google Scholar: 16  Indexed-papers identified by reviewing the reviews: 31 |
| **Keywords:** intercultural, salud, saúde, materna |
