# Supplementary material for: Navigating strategies for intercultural maternal and newborn care in Latin America and the Caribbean: a scoping review
Source: Health Promot Int. 2026 Jun 15;41(3):daag082. doi: 10.1093/heapro/daag082 (PMC13267143; doi:10.1093/heapro/daag082)
Supplement: daag082_Supplementary_Data [file daag082_supplementary_data.zip › Supplementary File 4_Characteristics of included studies.docx]

**Supplementary File 4:** Characteristics of included studies

| **Author year** | **Country** | **Population/**  **Groups addressed** | **Strategies to promote intercultural maternal and newborn care** |
| --- | --- | --- | --- |
| Abrams 2018 [1] | Haiti | Rural community | **Culturally relevant curriculum for pregnant women (Group Prenatal Care):** Fanm Pale (Women Speak) is a six-session intervention that integrates antenatal assessments with education and community building while addressing pregnancy-related topics **(I).** Integrate local cultural practices such as traditional prayers and songs into the curriculum **(P).**  **Language accessibility:** Sessions are conducted in English with Haitian Creole translation to promote inclusive participation **(I).** Implementing the programme in participants’ native language may improve outcomes **(P)**.  **Native advisory team:** Involve native advisors in programme planning to ensure cultural relevance and appropriateness **(P).** |
| Atyeo 2017 [2] | Guatemala | Indigenous | **Culturally competent breastfeeding education:** Integrating cultural beliefs into health education programmes. The authors found that mothers understood concepts like passive immunity and the antimicrobial benefits of human milk through culturally familiar terms, such as “cleaning the baby’s stomach” and “providing vitamins for the baby” **(P)**. |
| Austad 2020 [3] | Guatemala | Indigenous | **Obstetric Care Navigators (OCNs):** Trained Maya women act as OCNs to facilitate referrals from TBAs to public hospitals. They provide guidance and coordinate maternal care, combining elements of care navigation and doula support models **(I).** |
| Avellaneda 2015 [4] | Brazil | Bolivian immigrant women | Implement intercultural childbirth practices **(P).**  **Culturally appropriate services:** Provide tailored services and facilities, such as culturally sensitive birthing rooms **(P).**  **Training for biomedical staff:** Train healthcare professionals in intercultural practices and cultural traditions **(P)**. |
| Bautista‐Valarezo 2022 [5] | Ecuador | Indigenous | **Culturally relevant TBA training:** Culturally adapted flowcharts were co-developed with TBAs to enhance clinical responses to obstetric emergencies **(I)**. |
| Blas 2023 [6] | Peru | Indigenous | **Culturally relevant training to mothers and TBAs:** the *Mamás del Río* Programme consists of tablet-enhanced educational home visits by CHW to pregnant women and mothers of newborns, with training workshops on essential newborn care of TBAs and facility staff **(I)**. |
| Cañuta 2017 [7] | Chile | Indigenous | **Holistic or intercultural hospitals:** Example - Intercultural Hospital *Kalvu Llanka de Cañete* **(I).**  Train biomedical staff in intercultural practices and cultural traditions **(I)**.  *Casa de acogida*: Provide accommodation for individuals from remote areas, allowing them to stay overnight before early morning exams or procedures **(I).**  Implement intercultural childbirth practices **(I)**  **Culturally appropriate services:** Provide tailored services and facilities, such as culturally sensitive birthing rooms **(I).** |
| Cardona-Arias 2015 [8] | Colombia | Indigenous | **Dialogue between biomedical and traditional healthcare providers:** Facilitate shared decision-making between Western and traditional medicine practitioners to improve integrated care **(I)**. |
| Castillo-Santana 2017 [9] | Colombia | Indigenous | **Build a pluralistic health system:** Establish dialogue platforms mediated by Indigenous and Western health authorities to recognise and understand the logic of each health system **(P)**.  **Training biomedical staff:** Integrate practical and conceptual elements of interculturality into health sciences curricula and training **(P)**.  **Foster international cooperation:** Facilitate experience exchange in intercultural childbirth care with countries like Peru and Bolivia **(P)**.  **Translate policy into practice:** Ensure Indigenous peoples access culturally appropriate health services that comply with national regulations **(P)**. |
| Chary 2013 [10] | Guatemala | Indigenous | **Language accessibility:** Guatemalan midwife training programmes can be enhanced by providing instruction in local languages like Kaqchikel **(P)**.  **TBA training:** The expanding allopathic knowledge of Indigenous midwives can foster productive partnerships between lay midwives and the allopathic medical community **(P)**. |
| Chia López 2019 [11] | Peru | Indigenous | Implement intercultural childbirth practices **(I)**.  **Translate policy into practice:** fully implement the intercultural health policy **(P).** |
| Chomat 2014 [12] | Guatemala | Indigenous | **Language accessibility:** increase the number of health staff that speak Mayan languages **(P).**  **Training biomedical staff:** train healthcare staff to improve cultural competence and awareness of the value of traditional health systems **(P).**  Implement **intercultural childbirth practices** **(P).**  **Build a pluralistic health system** through dialogue, cultural exchange, and awareness **(P).**  Listen to Indigenous perspectives and address their priorities and needs **(P).**  **Establish a diverse and culturally sensitive health workforce:** increase the number of healthcare professionals sensitive to Indigenous norms and practices **(P)** |
| Chopel 2013 [13] | Mexico | Indigenous | **Holistic or intercultural hospitals:** Promote holistic hospitals (*hospitales integrales*) in Chihuahua to improve the desirability of hospital births **(P)**. |
| Coast 2014 [14] | Multiple countries: Argentina, Bolivia, Brazil, Cuba, Ecuador, Guatemala, Mexico, Peru | Indigenous and other groups | The review encompassed studies from 35 countries. In Latin America, it included 13 studies: three from Bolivia, two from Peru, one from Ecuador, three from Guatemala, two from Mexico, one covering Argentina, Brazil, Cuba, and Mexico, and one including Bolivia and Guatemala alongside non-Latin American countries. The reported strategies to promote intercultural maternal care included:  **Promote intercultural childbirth practices**, including family and TBAs **(I).**  **Training health workers to improve intercultural care** **(I).**  **Training of traditional birth attendants (I).**  **Provide culturally acceptable services** appropriate to women’s needs, including equipment to support vertical delivery positions (e.g., ropes and benches) **(I).**  **Encourage midwives to use their knowledge** **(I).**  Integration of an intercultural focus into **health policies (I).**  Establish culturally appropriate/intercultural **birth houses** **(I).**  Implement health education interventions and **participatory women’s groups** **(I).** |
| Coast 2016 [15] | Peru | Indigenous peoples and communities in high-risk districts | **Language accessibility:** Use of the Quechua language **(I).**  Implement intercultural childbirth practices **(I).**  Cultural adaptation of birthing services **(I)**  **Establish a diverse and culturally sensitive health workforce:** health professionals respectful of culture (I). |
| Cruz Llumiquinga 2021 [16] | Ecuador | Indigenous | **TBA training** **(I)**.  Implement intercultural childbirth practices **(I)**. |
| Del Mastro 2021 [17] | Peru | Indigenous | **Culturally relevant training to mothers and TBAs:** the *Mamás del Río* Programme **(I)**.  Replicate the Mamas del Rio programme nationwide **(P).**  Conduct formative research to build programmes **(I)**.  Implement intercultural childbirth practices **(P)**.  Cultural adaptation of birthing services **(P)**. |
| Fahey 2013 [18] | Guatemala | Indigenous | **Culturally relevant TBA training:** Adapt and implement PRONTO provider training by integrating *comadronas* into the scenarios **(I).** |
| Freitas Júnior 2018 [19] | Brazil | Quilombola women | **Training healthcare students:** Integrate Intercultural Health education into healthcare curricula, including cultural competence training focused on the care of Quilombola women **(I).** |
| Gallegos 2017 [20] | Ecuador | Indigenous | **Intercultural health office** in the Ministry of Public Health **(I).**  **Technicians in Primary Health Care:** Residents with two years of training serve as health promoters and intermediaries between public health professionals and the local population. In predominantly Indigenous areas, they speak the local language and understand cultural health beliefs and practices **(I).**  Integrate TBAs into the public health system **(P).**  Build a pluralistic health system **(PI).**  Translate policy into practice **(P).**  Cultural adaptation of birthing services **(I).** |
| Garcia 2018 [21] | Guatemala | Indigenous | **TBA Training on Obstetrical Emergencies:** Training TBAs to recognise emergencies using Take Action Cards from the Home Based Life Saving Skills curriculum by the American College of Nurse Midwives **(I).**  **Language accessibility:** conduct training in the native language of TBAs, respecting local practices **(I).** |
| Guerra-Reyes 2016 [22] | Peru | Indigenous | **Implement intercultural childbirth practices (I).**  **Culturally adapted services (I).**  **Widely implement the Intercultural Birthing Policy (P).**  **Training biomedical staff:** strengthen training programmes from an intercultural approach **(P).**  Establish a diverse and culturally sensitive health workforce **(P).** |
| Guerra-Reyes 2019 [23] | Peru | Indigenous | Implement intercultural childbirth practices (I).  Culturally adapted services (I).  Intercultural Birthing Policy (IBP) **(I).**  Maternal Waiting House (*Mama Wasi*) **(I).**  **Training healthcare students:** Anthropological engagement with intercultural health in medical education **(P).** |
| Gusman 2015 [24] | Brazil | Indigenous | **Empower, recognise, support, and integrate TBAs into the health system:** the Working with Traditional Midwives Programme by the Ministry of Health integrates TBAs into local health services, particularly family health teams, and recognises their roles. Efforts include surveying regional home births, registering active TBAs, and distributing basic childbirth kits **(I)**.  **TBA Training:** Pediatricians authorised by the Brazilian Society of Pediatrics (SBP) provide capacity-building on medical equipment use and neonatal resuscitation **(I).** |
| Hernandez 2017 [25] | Guatemala | Indigenous | **Culturally relevant TBA training on obstetric emergencies:** the School of POWHER Training Programme focuses on training *comadronas* in maternal healthcare while respecting local customs and appropriate obstetric referrals **(I)**.  **TBAs as trainers:** train *Comadronas* become future trainers for the programme **(I)**. |
| Hernandez 2018 [26] | Guatemala | Indigenous | **Language accessibility:** programme delivered in Spanish and Tz’tujil (a local Mayan language) **(I)**.  **Culturally relevant TBA training:** The School of POWHER training is an immersive programme respecting cultural Mayan practices and emphasizing signs of referrals for the mom and baby, prenatal care, and initial management of postpartum complications **(I)**.  **TBA support:** Each woman who completes the School of POWHER training programme receives a stethoscope, blood pressure equipment, a fetal Doppler, prenatal vitamins, and safe birthing kits **(I).** |
| Hurtado Zambrana 2014 [27] | Bolivia | Indigenous and other vulnerable communities | Train healthcare personnel in intercultural maternal health topics **(P)**.  Train students in intercultural health topics **(P)**.  Promote broad discussions on intercultural health with the general public **(P)**. |
| Ibáñez-Cuevas 2015 [28] | Mexico | Indigenous | Implement intercultural childbirth practices **(I)**.  Maternity Homes **(I)**.  **Language accessibility:** Institutions should have staff capable of communicating with users in their native language while ensuring that the information provided retains the accuracy intended by doctors **(P)**.  Implement or strengthen the Project *Casa de la Mujer Indígena* or *Casas* Model. It is an initiative that unites the local Indigenous community, NGOs, and public institutions to create a space to improve health care and patient satisfaction for Indigenous women **(P)**. |
| Kestler 2020 [29] | Guatemala | Indigenous | **Culturally relevant social marketing campaign** to encourage pregnant women to give birth in their closest health centre **(I).**  **Culturally relevant TBA training** focused on obstetric and neonatal emergencies. The PRONTO provider training is a low-cost simulation using PartoPants to teach TBAs emergency management, teamwork, and communication **(I).**  **TBA recognition, support, and integration into the healthcare system:** outreach activities by professional midwives to improve the link between TBAs and the formal healthcare system **(I).** |
| Lazo-Gonzales 2023 [30] | Peru | Indigenous | Maternal Waiting Houses **(PI)**.  Implement intercultural childbirth practices **(PI)**. |
| Llamas 2016 [31] | Ecuador | Indigenous | Implementing vertical births as an **intercultural health policy** in public institutions **(I)**.  **Training biomedical staff:** train health professionals to deliver women in vertical positions **(I)**.  **TBA recognition, support, and integration into the healthcare system:** provide TBAs with training and equipment and strengthen the links between the Ministry of Health and the TBAs **(I)**. |
| Llamas 2018 [32] | Ecuador | Indigenous | Implementing a vertical birth policy in a hospital **(I)**.  **Training biomedical staff:** healthcare workers received training on Kichwa (the local indigenous language), Indigenous culture, and clinical issues such as delivering women in upright positions **(I)**. |
| Matute 2021 [33] | Ecuador | Indigenous and mestizo | Implement intercultural childbirth practices **(I)**.  **Training biomedical staff** to incorporate cultural practices in maternal care **(I)**. |
| Miller 2017 [34] | Multiple countries: Peru, Mexico, Guatemala, Bolivia | Indigenous and other groups | **Language accessibility:** use of the Quechua language **(I)**.  Implement intercultural childbirth practices **(I)**.  Implement culturally adapted birthing rooms **(I)**.  TBA recognition, support, and integration into the formal healthcare system **(I)**.  Establish a diverse and culturally sensitive health workforce: health professionals respectful of culture **(I)**. |
| Moore 2018 [35] | Peru | Indigenous, rural and other communities | Implementation of Intercultural Health Policy: *Plan Estratégico de Salud Sexual y Reproductiva* **(I)**.  Maternal Waiting Houses (Casas de Espera Materna) **(I)**.  Implement intercultural childbirth practices, including vertical birth **(I)**.  Cash conditionality programmes such as Juntos – rewarding women with a cash allowance in exchange for their adherence to maternal health policy – are used to encourage their participation **(I)**. |
| Morales 2018 [36] | Bolivia | Indigenous | Implement intercultural childbirth practices **(I)**  Implement culturally adapted birthing rooms in a hospital **(I)**. |
| Nacht 2022 [37] | Guatemala | Rural community | **TBA recognition and integration:** Antenatal care is provided by skilled nurses along with TBAs **(PI)**. |
| Olaza-Maguiña 2024 [38] | Peru | Indigenous | **Translate policy into practice:** Real application of intercultural approach and humanised birth care, with active participation of women **(P)**. |
| Olivas 2023 [39] | Guatemala | Indigenous | **Maternity homes (*Casas maternas* or Community Birthing Centres):** Community-established, owned, and operated birthing centres where auxiliary nurses deliver accessible and culturally appropriate care **(I)**. |
| Ortiz 2022 [40] | Colombia | Indigenous | **Culturally relevant Kangaroo-Mother training:** Adaptations of the Kangaroo-Mother method (KMM) at home and care practices, protection and healing based on customs, and cultural tradition. Promote synergy between knowledge acquired from nurses at neonatal ICU during KMM training and traditional knowledge **(I).** |
| PAHO 2023 [41] | Multiple countries | Indigenous peoples, Afro-descendants, Roma, and the members of other ethnic groups | **Implement evaluation tool:** Excel tool for determining whether maternal health services have adopted an intercultural approach. This tool evaluates childbirth care from the perspectives of health providers, Indigenous women, and external observers across five key dimensions: autonomy, communication, community participation, infrastructure (including culturally appropriate maternity homes), and institutional strengthening **(P).** |
| Penn-Kekana 2017 [42] | Multiple countries: Honduras, Peru, Nicaragua, Cuba, Guatemala | Indigenous and rural communities | Maternity Waiting Homes (I) |
| Perge 2021 [43] | Haiti | High-risk communities | **TBA recognition:** Encourage TBAs (matrons) to refer pregnant women to prenatal care at health facilities by awarding them a plaque of honor signed by the facility doctor. Matrons who successfully advise women to attend prenatal care can also participate in a lottery to win a small token of appreciation **(P)**.  **Overcome optimism bias:** 10-minute video for pregnant women and matrons with testimonials from mothers, matrons, nurses, and community health workers recognizing the importance of prenatal care and highlighting the potential risks **(P)**. |
| Pulido Hernández 2022 [44] | Mexico | Indigenous | **New professionals and dialogue:** Intercultural interlocutors, indigenous interpreter-translators, and intercultural facilitators can foster dialogical intercultural communication in healthcare to achieve mutual understanding **(I)**. |
| Radoff 2013 [45] | Nicaragua | Indigenous | **Language accessibility:** chapters in Spanish and Miskito **(I).**  **Culturally appropriate, community-based radio soap opera to improve women’s knowledge of pregnancy danger signs**, promote rapid referral, and encourage facility-based births. Each episode closes with a review of danger signs and a Maternity House promotion **(I)**. |
| Rangel Flores 2017 [46] | Mexico | Indigenous | **Training biomedical staff:** strengthen training programmes from an intercultural approach **(P).**  **Dialogue between biomedical and traditional healthcare providers:** sensitise stakeholders to respect other forms of knowledge **(P)**. |
| Rojas 2015 [47] | Peru | Indigenous | Implement intercultural health services: infrastructure, healthcare staff, and equipment **(P).**  Training biomedical staff in intercultural maternal health and local cultural beliefs and practices **(P).**  TBA integration: Strengthen the collaboration between biomedical staff and TBAs **(P).**  Translate policy into practice **(P).** |
| Roosta-G 2015 [48] | Bolivia | Indigenous | **Implement intercultural childbirth practices (I)**  **Culturally appropriate services and installations:** (1) Room for the integration of traditional and Western medicine in a hospital; (2) Culturally adapted birthing rooms have been implemented, side by side with the biomedical room, making possible their articulation and complementation **(I)**.  **Foster research:** Generate knowledge on medicinal plants **(I).** |
| Ruiz 2013 [49] | Guatemala | Indigenous | **Maternity waiting homes:** Implement culturally appropriate maternity waiting homes with community involvement and support through a participatory approach (interactive workshops) involving Mayan women and TBAs **(P)**.  Implement communication strategies to increase awareness about maternity homes **(P)**. |
| Samuel 2016 [50] | Peru | Indigenous | Implement a **citizen monitoring** initiative to improve the adherence of healthcare services to the intercultural birthing policy **(I)**. |
| Santana Mera 2016 [51] | Ecuador | Indigenous and afro-descendants in rural or urban areas, vulnerable communities | Implement **culturally relevant TBA training** near their homes **(P).**  TBA effective **integration** into the formal healthcare system **(P).** |
| Sarmiento 2020a [52] | Mexico | Indigenous | **Research as strategy:** Participatory research and intercultural dialogue as strategies to improve maternal health with Indigenous communities in Mexico’s Guerrero State **(I)**. |
| Sarmiento 2020b [53] | Mexico | Indigenous | **Research as strategy:** Mapping Indigenous knowledge to incorporate participant voices into research and decision-making in maternal health **(I).** |
| Sarmiento 2021 [54] | Mexico | Indigenous | **Research as strategy:** Intercultural researchers can provide a substrate for intercultural dialogue in maternal health through literature review and stakeholder maps **(I)**. |
| Sarmiento 2022b [55] | Mexico | Indigenous | **Material support to TBAs:** Traditional midwives received stipends to dedicate more time to patient care **(I).**  **Apprentice support to TBAs:** Each midwife appointed an apprentice, who received a scholarship and helped with tasks the midwives could no longer perform due to age **(I)**.  **Training biomedical staff:** Researchers conducted workshops in local health centres, highlighting the role of traditional midwives and the need for intercultural skills in Western medical practice.  **Intercultural health brokers:** Bilingual community members trained as intercultural health brokers support traditional midwives and improve collaboration with Western healthcare providers **(I)**. |
| Sarmiento 2022 [56] | Mexico | Indigenous | **Intercultural health brokers:** Training community members as intercultural brokers to help them recognise the role of traditional midwives and the value of their own culture **(I)**.  **TBA training** organised by the government **(I)**.  Intercultural health practices: TBA and intercultural broker present during childbirth **(I)**. |
| Solís 2023 [57] | Chile | Indigenous | Implement intercultural childbirth practices **(P)**.  Implement intercultural health services: infrastructure, healthcare staff, and equipment **(P).**  Dialogue between biomedical and traditional healthcare providers **(P).** |
| Sousa 2019 [58] | Guatemala | Indigenous | Language accessibility **(I)**.  Provide culturally relevant training for TBAs and other professionals in basic life support and ultrasound **(I)**. |
| Stollak 2016 [59] | Guatemala | Indigenous | **Implement Maternity Houses (*Casas Maternas*) (I)**.  An obstetric nurse provides regular monthly training to the Casa Materna staff and continuous monitoring of the quality of their work **(I)**. |
| Stroux 2016 [60] | Guatemala | Indigenous | **Language accessibility:** audio instructions recorded in the Mayan dialect Kaqchikel by Wuqu' Kawoq staff and TBAs **(PoC)**.  **Culturally relevant TBA training: a** mHealth Monitoring System to train traditional birth attendants to assess perinatal risk early, reducing delays in advice seeking and integrating them into the referral process. Illustrations tailored to the local context and photos staged to ensure cultural appropriateness **(PoC)**. |
| Suárez-Baquero 2021 [61] | Multiple countries | Indigenous and afro-descendant | **TBA inclusion** into the formal health care systems **(P)**  Establish **associations** for TBAs (Mexico and Colombia) **(I)**  Formal documentation of births and maternal outcomes by TBAs **(P)**  TBA support and recognition by **policymakers (P).** |
| Summer 2017 [62] | Guatemala | Indigenous | Implement a **professional midwifery school** accredited by the government to increase skilled birth attendants in rural and indigenous communities **(PP)**.  Professional midwives train and work together with TBAs **(P).**  **Reintroduce the professional midwife into the health system**, coordinating with community leaders, local representative bodies, mayors, and health commissions **(PP)**. |
| Summer 2018 [63] | Guatemala | Indigenous and rural communities | Implement a government-sponsored **professional midwifery school** exclusively for Indigenous women from rural Huehuetenango who speak a Mayan language **(PP).** |
| Summer 2019 [64] | Guatemala | Indigenous and rural communities | Create the professional midwifery profession in Guatemala **(I).**  TBA inclusion in the health system **(PI).**  Integration of intercultural childbirth practices in the health facilities **(I).** |
| Torri 2013a [65] | Bolivia | Indigenous | Language accessibility: Quechua-speaking personnel **(I)**.  Implement intercultural childbirth practices **(I)**  **Cultural adaptation of birthing facilities**  Train biomedical staff in Andean health and sickness concepts, ethno-anatomy, and ethno-physiology **(I)**.  TBA integration into the health services **(I).** |
| Torri 2013b [66] | Ecuador | Indigenous | Create an herbal garden near the hospital to cultivate medicinal plants that TBAs use for reproductive health **(I)**.  **Dialogue:** Knowledge exchange, especially with the nurses between indigenous medicine and Western Europe **(I)**.  **Research:** Analysis of the possible dangers and benefits of the different traditional therapeutic practices to develop medical pluralism **(P)**.  **Training biomedical professionals** in ethnobotanical and ethnopharmacological dimensions of medicinal plants used by TBAs **(P)**. |
| Tovilla 2022 [67] | Mexico | Indigenous | Implement an intercultural birthing room in a health service **(I).** |
| Trejos Serrato 2022 [68] | Colombia | Indigenous | Implement intercultural childbirth practices **(I)** |
| Tucker 2013 [69] | Mexico | Indigenous | Maternity House or Intercultural birthing house (*Casa Materna*) programme **(I)**. |
| UNICEF Argentina 2023 [70] | Argentina | Indigenous | **Implement intercultural childbirth practices**, including rooms with appropriate equipment **(I).**  **Language Accessibility:** training bilingual facilitators to help in the accompaniment of people from native communities and provide educational activities in local languages **(I).**  Training healthcare personnel **(I).**  Incorporate the perspective of chiefs and community leaders in intercultural maternal health **(I).**  Promote workshops on sexual and reproductive health for adolescents and young mothers **(I).** |
| van Braam 2023 [71] | Multiple countries: Guatemala, Nicaragua | Rural community | Maternity Waiting Houses |
| van Dijk, 2013 [72] | Guatemala | Indigenous | **Language Accessibility:** provide care in the woman's language **(PI).**  Implement intercultural childbirth practices **(PI).**  **Training biomedical personnel** on local obstetric practices, cultural sensitivity, TBA integration in maternal care services, and vertical birthing techniques **(PI).** |
| Vicente Martín 2017 [73] | Bolivia | Indigenous | Implement intercultural childbirth practices **(I)**  Implement culturally adapted birthing rooms **(I)**  Translate policy into practice **(P)** |
| Walsh 2017 [74] | Haiti | Rural community | **Empowering TBAs to implement campaigns:** TBAs trained to apply chlorhexidine to the umbilical cord and teach mothers to use it instead of unhygienic substances **(I)**;  **Recognise strong cultural beliefs and provide culturally sensitive recommendations.** For example, given the strong belief in Haiti that covering the cord is essential for newborn protection, providing clean, porous gauze at birth could respect these cultural practices while keeping the cord site clean and dry **(P)**. |
| Walton 2016 [75] | Guatemala | Indigenous | **Culturally relevant TBA training:** PRONTO provider training implementation, a low-technology simulation training scheme for obstetric and perinatal emergency management **(I)**. |

CHW: Community health workers; TBA: traditional birth attendant; P: proposed*; PP: proposed and planned*; I: Implemented*; PI: Partially implemented*; PoC: Proof of Concept*

*At the date of study publication

**References**

1. Abrams JA, Forte J, Bettler C, Maxwell M. Considerations for Implementing Group-Level Prenatal Health Interventions in Low-Resource Communities: Lessons Learned From Haiti. J Midwifery Womens Health 2018;63:121–6.
2. Atyeo NN, Frank TD, Vail EF, *et al*. Early Initiation of Breastfeeding Among Maya Mothers in the Western Highlands of Guatemala: Practices and Beliefs. J Hum Lact 2017;33:781–9.
3. Austad K, Juarez M, Shryer H, *et al.* Obstetric care navigation: results of a quality improvement project to provide accompaniment to women for facility-based maternity care in rural Guatemala. BMJ Qual Saf 2020;29:169–78.
4. Avellaneda Yajahuanca RdS. A experiência de gravidez, parto e pós-parto das imigrantes bolivianas e seus desencontros na cidade de São Paulo - Brasil [thesis]. São Paulo: Universidade de ​São Paulo; 2015.
5. Bautista-Valarezo E, Espinosa ME, Michels NRM, *et al*. Culturally adapted flowcharts in obstetric emergencies: a participatory action research study. BMC Pregnancy Childbirth 2022;22:772.
6. Blas MM, Reinders S, Alva A, *et al*. Effect of the Mamás del Río programme on essential newborn care: a three-year before-and-after outcome evaluation of a community-based, maternal and neonatal  health intervention in the Peruvian Amazon. Lancet Reg Health Am 2023;28:100634.
7. Cañuta CA. Las múltiples paradojas de la política de salud intercultural en la atención de salud materna que promueve el estado chileno. El caso del Hospital Kalvu Llanka de Cañete [thesis]. Oaxaca: Centro de Investigaciones y Estudios Superiores en Antropología Social; 2017.
8. Cardona-Arias JA, Rivera-Palomino Y, Carmona-Fonseca J. Expresión de la interculturalidad en salud en un pueblo emberá-chamí de Colombia. Rev Cuba Salud Pública 2015;41:77-93
9. Castillo-Santana PT, Vallejo-Rodríguez ED, Cotes-Cantillo KP, *et al*. Salud materna indígena en mujeres Nasa y Misak del Cauca, Colombia: tensiones, subordinación y diálogo intercultural entre dos sistemas médicos. Saúde e Soc 2017;26:61–74.
10. Chary A, Díaz AK, Henderson B, *et al*. The changing role of indigenous lay midwives in Guatemala: new frameworks for analysis. Midwifery 2013;29:852–8.
11. Chia López SI, Diaz Herrera A. Implementación de la política sectorial de salud intercultural, relacionada a la salud materna neonatal, en mujeres quechuas de 15 a 49 años, del distrito de Vilcashuamán y Saurama, provincia de Vilcashuamán, departamento de Ayacucho 2018 [thesis]. Lima: Pontificia Universidad Católica de Perú; 2019.
12. Chomat AM, Solomons NW, Montenegro G, *et al*. Maternal health and health-seeking behaviors among indigenous Mam mothers from Quetzaltenango, Guatemala. Rev Panam Salud Publica 2014;35:113–20.
13. Chopel AM. Reproductive health in indigenous Chihuahua: giving birth ‘alone like the goat’. Ethn Health 2013;19:270–96.
14. Coast E, Jones E, Portela A, *et al*. Maternity care services and culture: a systematic global mapping of interventions. PloS One 2014;9:e108130. doi: 10.1371/journal.pone.0108130.
15. Coast E, Jones E, Lattof SR, *et al*. Effectiveness of interventions to provide culturally appropriate maternity care in increasing uptake of skilled maternity care: a systematic review. Health Policy Plan 2016;31:1479–91.
16. Cruz Llumiquinga FE. Impacto de la salud materna con enfoque intercultural en el desarrollo humano de mujeres indígenas de la provincia de Imbabura entre el año 2008 a 2018 [thesis]. Buenos Aires: Facultad Latinoamericana de Ciencias Sociales Argentina; 2021.
17. Del Mastro N I, Tejada-Llacsa PJ, Reinders S, *et al*. Home birth preference, childbirth, and newborn care practices in rural Peruvian Amazon. PloS One 2021;16:e0250702. doi: 10.1371/journal.pone.0250702
18. Fahey 2013Fahey JO, Cohen SR, Holme F, *et al*. Promoting cultural humility during labor and birth: putting theory into action during PRONTO obstetric and neonatal emergency training. J Perinat Neonatal Nurs 2013;27:36–42.
19. Freitas Júnior RA de O, Santos CAD, Lisboa LL, *et al*. Incorporando a Competência Cultural para Atenção à Saúde Materna em População Quilombola na Educação das Profissões da Saúde. Rev Bras Educ Médica 2018;42:100-109.
20. Gallegos CA, Waters WF, Kuhlmann AS. Discourse versus practice: are traditional practices and beliefs in pregnancy and childbirth included or excluded in the Ecuadorian health care system? Int Health 2017;9:105–11.
21. Garcia K, Dowling D, Mettler G. Teaching Guatemalan traditional birth attendants about obstetrical emergencies. Midwifery 2018;61:36–8.
22. Guerra-Reyes L. Implementing a culturally appropriate birthing policy: Ethnographic analysis of the experiences of skilled birth attendants in Peru. J Public Health Policy 2016;37:353–68.
23. Guerra-Reyes L. Numbers that Matter: Right to Health and Peruvian Maternal Strategies. Med Anthropol 2019;38:478–92.
24. Gusman CR, Viana APdAL, Miranda MAB, *et al.* Inclusion of traditional birth attendants in the public health care system in Brazil: reflecting on challenges. Rev Panam Salud Publica. 2015;37:365–70.
25. Hernandez S, Oliveira JB, Shirazian T. How a Training Program Is Transforming the Role of Traditional Birth Attendants from Cultural Practitioners to Unique Health-care Providers: A Community Case Study in Rural Guatemala. Front Public Health 2017;5:111.
26. Hernandez S, Oliveira J, Jones L, *et al*. Impact of Standardized Prenatal Clinical Training for Traditional Birth Attendants in Rural Guatemala. Healthcare (Basel) 2018;6:60.
27. Hurtado Zambrana JL, Manjón Calvimontes N, Pérez Mendoza R, et al. Factores relacionados a la atención con enfoque intercultural en los servicios de salud materna sucre, 2009. ENFERvida. 2014;2:8–16.
28. Ibáñez-Cuevas M, Heredia-Pi IB, Meneses-Navarro S, *et al*. Labor and delivery service use: indigenous women’s preference and the health sector response in the Chiapas Highlands of Mexico. Int J Equity Health 2015;14:156.
29. Kestler E, Ambrosio G, Hemming K, *et al*. An integrated approach to improve maternal and perinatal outcomes in rural Guatemala: A stepped-wedge cluster randomized trial. Int J Gynaecol Obstet Off Organ Int Fed Gynaecol Obstet 2020;151:109–16.
30. Lazo-Gonzales AO, Sarmiento-Casavilca T, Espinosa-Henao OE, *et al*. Looking at maternal health of Asháninka communities from the conceptual framework of the accessibility of care. Int J Equity Health 2023;22:154.
31. Llamas A, Mayhew S. The emergence of the vertical birth in Ecuador: an analysis of agenda setting and policy windows for intercultural health. Health Policy Plan 2016;31:683–90.
32. Llamas A, Mayhew S. ‘Five hundred years of medicine gone to waste’? Negotiating the implementation of an intercultural health policy in the Ecuadorian Andes. BMC Public Health 2018;18:686.
33. Matute SED, Martinez EZ, Donadi EA. Intercultural Childbirth: Impact on the Maternal Health of the Ecuadorian Kichwa and Mestizo People of the Otavalo Region. Rev Bras Ginecol Obstet 2021;43:14–9.
34. Miller T, Smith H. Establishing partnership with traditional birth attendants for improved maternal and newborn health: a review of factors influencing implementation. BMC Pregnancy Childbirth 2017;17:365.
35. Moore MA. Interculturality from Below: An Ethnography of Maternal Health Encounters in the Peruvian Andes [thesis]. Liverpool: The University of Liverpool; 2018.
36. Morales GE. There Is No Place Like Home: Imitation and the Politics of Recognition in Bolivian Obstetric Care. Med Anthropol Q 2018;32:404–24.
37. Nacht A, Rivera C, Montes SB, *et al*. The Addition of Traditional Birth Attendant Care to a Home-Based Skilled Nursing Program in Rural Guatemala: A Secondary Analysis from a Quality Improvement  Database. J Midwifery Womens Health 2022;67:107–13.
38. Olaza-Maguiña AF, De La Cruz-Ramirez YM. Factors associated with negative birth experience in Peruvian Quechua-speaking indigenous women in a context of contagion due to COVID-19. Int J Gynaecol Obstet 2024;164:633–40.
39. Olivas ET, Valdez M, Muffoletto B, *et al*. Reducing inequities in maternal and child health in rural Guatemala through the CBIO+ Approach of Curamericas: 6. Management of pregnancy complications at  Community Birthing Centers (Casas Maternas Rurales). Int J Equity Health. 2023;21(Suppl 2):204.
40. Ortiz Anaya Y, Guillermo Rojas J. Cultural Care Practices Provided at Home by the Zenú Indigenous Mothers to their Premature Children and to Those with Low Birth Weight. Investig Educ En Enfermeria. 2022;40:e09. doi.org/10.17533/udea.iee.v40n2e09.
41. Pan American Health Organization. Tool for Promoting Culturally Safe Childbirth: Basic Manual. 2023. Available: https://iris.paho.org/handle/10665.2/57116 [Accessed 10 Jan 2025]
42. Penn-Kekana L, Pereira S, Hussein J, *et al*. Understanding the implementation of maternity waiting homes in low- and middle-income countries: a qualitative thematic synthesis. BMC Pregnancy Childbirth 2017;17:269.
43. Perge EB, Llopis Abella. A Behavioral Approach to Uncover Barriers to Maternal Care in Haiti. Washington, DC World Bank; 2021.
44. Pulido Hernández Y. Los espacios interculturales en salud materna: Una oportunidad para entablar el diálogo intercultural. Carta Tepa Mayo 4 2022;1:87–113.
45. Radoff KA, Levi AJ, Thompson LM. A radio-education intervention to improve maternal knowledge of obstetric danger signs. Rev Panam Salud Publica 2013;34:213–9.
46. Rangel Flores YY, Hernández Ibarra LE, González Acevedo CE, *et al*. Agenciamientos y resistencias en el cuidado obstétrico comunitario tras la capacitación institucional. Index Enferm 2017;26:250–4.
47. Rojas IC. Estudio del servicio de salud materna en el hospital general del distrito de Jaén, brindado a través del Seguro Integral de Salud: Análisis desde el enfoque intercultural durante los meses de marzo a setiembre de 2012 [thesis]. Lima: Pontificia Universidad Catolica del Peru; 2015.
48. Roosta-G. M. Madres indígenas enfrentan más de tres demoras: Los desafíos de la interculturalidad en salud. J Selva Andina Res Soc 2015;6:64–74.
49. Ruiz 2013Ruiz MJ, van Dijk MG, Berdichevsky K, *et al*. Barriers to the use of maternity waiting homes in indigenous regions of Guatemala: a study of users’ and community members’ perceptions. Cult Health Sex 2013;15:205–18.
50. Samuel J. The role of civil society in strengthening intercultural maternal health care in local health facilities: Puno, Peru. Glob Health Action 2016;9:33355. doi: 10.3402/gha.v9.33355
51. Santana Mera LJ. Estrategia de Integración al Sistema de Salud Pública Ecuatoriano de Parteras/Os, Parroquia Quisapincha, Ambato, 2016 [thesis]. Ambato: Universidad Técnica de Ambato; 2016.
52. Sarmiento I, Paredes-Solís S, Loutfi D, *et al*. Fuzzy cognitive mapping and soft models of indigenous knowledge on maternal health in Guerrero, Mexico. BMC Med Res Methodol 2020;20:125.
53. Sarmiento I, Zuluaga G, Paredes-Solís S, *et al*. Bridging Western and Indigenous knowledge through intercultural dialogue: lessons from participatory research in Mexico. BMJ Glob Health 2020;5:e002488. doi: 10.1136/bmjgh-2020-002488.
54. Sarmiento I, Paredes-Solís S, Dion A, Silver H, Vargas E, Cruz P, et al. Maternal health and Indigenous traditional midwives in southern Mexico: contextualisation of a scoping review. BMJ Open 2021;11:e054542. doi: 10.1136/bmjopen-2021-054542
55. Sarmiento I, Paredes-Solís S, de Jesús García A, Maciel Paulino N, Serrano de Los Santos FR, Legorreta-Soberanis J, et al. Safe birth in cultural safety in southern Mexico: a pragmatic non-inferiority cluster-randomised controlled trial. BMC Pregnancy Childbirth 2022;22:43.
56. Sarmiento I, Paredes-Solís S, De Jesús-García A, *et al*. Traditional Midwifery Contribution to Safe Birth in Cultural Safety: Narrative Evaluation of an Intervention in Guerrero, Mexico. Community Health Equity Res Policy 2024;44:377–89.
57. Solís MPA, Puschner SMC, Ibarra VV, *et al*. Experiencias locales de salud materna con perspectiva intercultural en Putre y Tirúa, Chile. Desacatos Rev Cienc Soc. 2023;71:130–45.
58. Sousa MF, Corning-Davis B. Building Capacity to Provide Maternal Health Care in an Indigenous Guatemalan Community Through Ultrasound and Skills Training. J Radiol Nurs 2019;38:123–30.
59. Stollak I, Valdez M, Rivas K, *et al*. Casas Maternas in the Rural Highlands of Guatemala: A Mixed-Methods Case Study of the Introduction and Utilization of Birthing Facilities by an Indigenous  Population. Glob Health Sci Pract 2016;4:114–31.
60. Stroux L, Martinez B, Coyote Ixen E, *et al*. An mHealth monitoring system for traditional birth attendant-led antenatal risk assessment in rural Guatemala. J Med Eng Technol 2016;40:356–71.
61. Suárez-Baquero DFM, Champion JD. Traditional partería providing women’s health care in Latin America: A qualitative synthesis. Int Nurs Rev 2021;68:533–42.
62. Summer A, Guendelman S, Kestler E, *et al*. Professional midwifery in Guatemala: A qualitative exploration of perceptions, attitudes and expectations among stakeholders. Soc Sci Med 2017 ;184:99–107.
63. Summer A, Walker D. Recommendations for Sustainable Midwifery in Guatemala. World Med Health Policy 2018;10:356–80.
64. Summer A, Walker D, Guendelman S. A Review of the Forces Influencing Maternal Health Policies in Post-War Guatemala. World Med Health Policy 2019;11:59–82.
65. Torri MC, Hollenberg D. Indigenous traditional medicine and intercultural healthcare in Bolivia: a case study from the Potosi region. J Community Health Nurs 2013;30:216–29.
66. Torri MC. Perceptions and uses of plants for reproductive health among traditional midwives in Ecuador: moving towards intercultural pharmacological practices. Midwifery 2013;29:809–17.
67. Tovilla GA. Impacto de la aplicación de la sala de atención al parto intercultural SNAIL BU CHVOK OLOL en las tasas de mortalidad materna del Centro de Salud CHIMIX DOS. Rev Latinoam Investig Educ. 2022;1:18–25.
68. Trejos Serrato J. Experiência da equipe de enfermagem no cuidado às mulheres indígenas no processo de parto e nascimento em Cauca, Colômbia [thesis]. Curitiba: Universidade Federal do Paraná; 2022
69. Tucker K, Ochoa H, Garcia R, *et al*. The acceptability and feasibility of an intercultural birth center in the highlands of Chiapas, Mexico. BMC Pregnancy Childbirth 2013;13:94.
70. UNICEF. Una iniciativa para proteger a las madres y sus bebés. UNICEF Argentina; 2023. Available: https://www.unicef.org/argentina/historias/salud-materna-intercultural#:~:text=La%20iniciativa%20comprende%20distintas%20instancias,embarazos%20no%20intencionales%20en%20adolescentes [Accessed 11 Jan 2025]
71. van Braam EJ, McRae DN, Portela AG, Stekelenburg J, Penn-Kekana L. Stakeholders’ perspectives on the acceptability and feasibility of maternity waiting homes: a qualitative synthesis. Reprod Health 2023;20:101.
72. van Dijk M, Ruiz MJ, Letona D, García SG. Ensuring intercultural maternal health care for Mayan women in Guatemala: a qualitative assessment. Cult Health Sex. 2013;15 Suppl 3:S365-382. doi: 10.1080/13691058.2013.779026.
73. Vicente Martín P. Los estudios de salud materna intercultural en Bolivia de la teoría a la praxis. Bol Am 2017;74:91–111.
74. Walsh SM, Norr KF, Sipsma H, *et al*. Effectiveness of a campaign to implement chlorhexidine use for newborns in rural Haiti. BMC Res Notes 2017;10:742.
75. Walton A, Kestler E, Dettinger JC, *et al*. Impact of a low-technology simulation-based obstetric and newborn care training scheme on non-emergency delivery practices in Guatemala. Int J Gynaecol Obstet 2016;132:359–64.
