# Supplementary material for: Navigating strategies for intercultural maternal and newborn care in Latin America and the Caribbean: a scoping review
Source: Health Promot Int. 2026 Jun 15;41(3):daag082. doi: 10.1093/heapro/daag082 (PMC13267143; doi:10.1093/heapro/daag082)
Supplement: daag082_Supplementary_Data [file daag082_supplementary_data.zip › Supplementary File 6_Summary of publication characteristics.pdf]

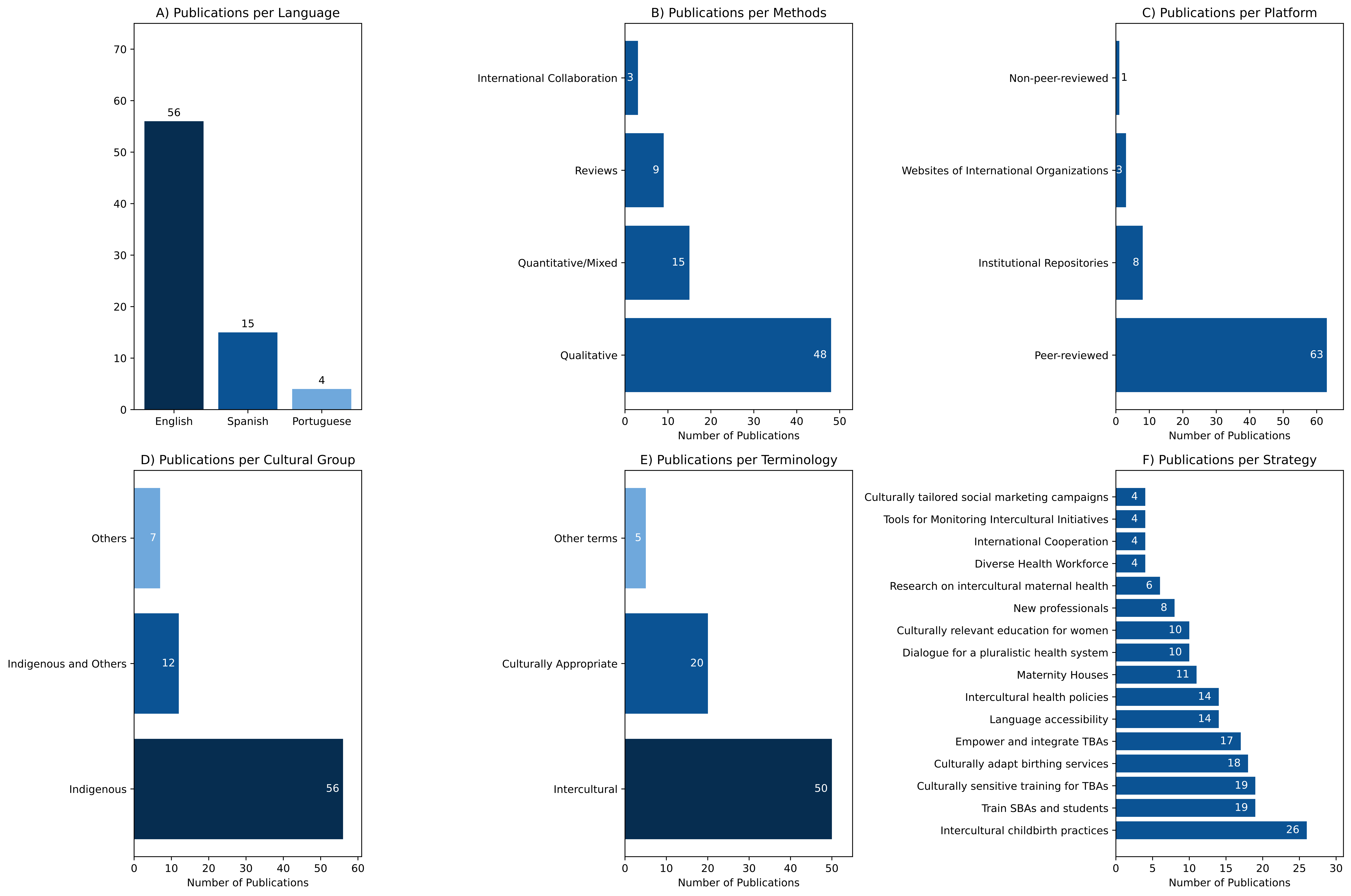
A) Publications per Language
56
15
4
English
Spanish
Portuguese
B) Publications per Methods
International Collaboration 3
Reviews 9
Quantitative/Mixed 15
Qualitative 48
Number of Publications
C) Publications per Platform
Non-peer-reviewed 1
Websites of International Organizations 3
Institutional Repositories 8
Peer-reviewed 63
Number of Publications
D) Publications per Cultural Group
Others 7
Indigenous and Others 12
Indigenous 56
Number of Publications
E) Publications per Terminology
Other terms 5
Culturally Appropriate 20
Intercultural 50
Number of Publications
F) Publications per Strategy
Culturally tailored social marketing campaigns 4
Tools for Monitoring Intercultural Initiatives 4
International Cooperation 4
Diverse Health Workforce 4
Research on intercultural maternal health 6
New professionals 8
Culturally relevant education for women 10
Dialogue for a pluralistic health system 10
Maternity Houses 11
Intercultural health policies 14
Language accessibility 14
Empower and integrate TBAs 17
Culturally adapt birthing services 18
Culturally sensitive training for TBAs 19
Train SBAs and students 19
Intercultural childbirth practices 26
Number of Publications
