# Supplementary figures and images for: Navigating strategies for intercultural maternal and newborn care in Latin America and the Caribbean: a scoping review
Source: Health Promot Int. 2026 Jun 15;41(3):daag082. doi: 10.1093/heapro/daag082 (PMC13267143; doi:10.1093/heapro/daag082)

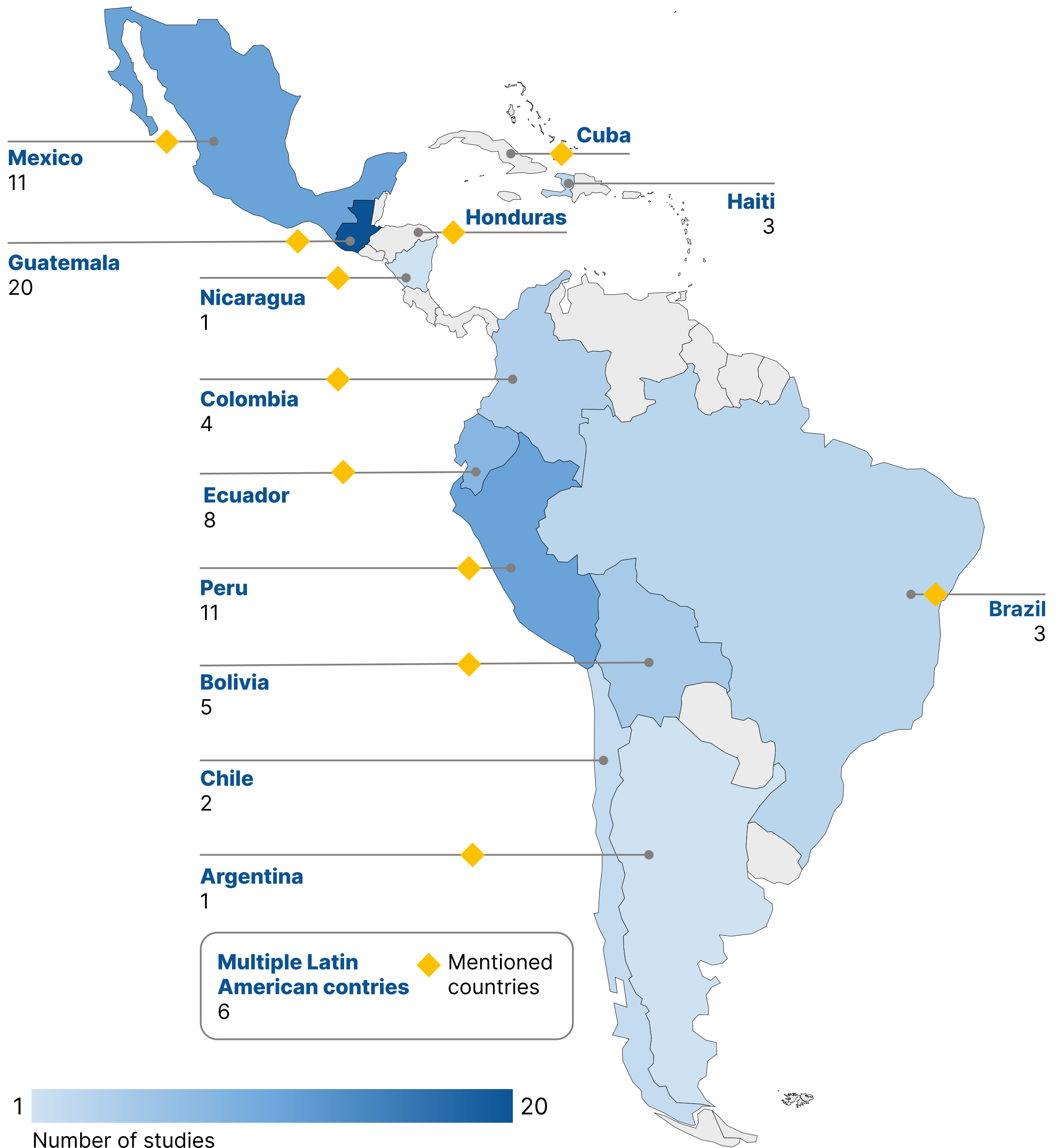

Supplement: daag082_Supplementary_Data [file daag082_supplementary_data.zip › Supplementary File 5_Geographical distribution of the studies.pdf]
